# Host Immune Cell Membrane Deformability Governs the Uptake Route of Malaria-Derived Extracellular Vesicles

**DOI:** 10.1021/acsnano.4c07503

**Published:** 2025-03-03

**Authors:** Daniel Alfandari, Irit Rosenhek-Goldian, Ewa Kozela, Reinat Nevo, Marcela Bahlsen Senprún, Anton Moisieiev, Noam Sogauker, Ido Azuri, Samuel Gelman, Edo Kiper, Daniel Ben Hur, Raviv Dharan, Raya Sorkin, Ziv Porat, Mattia I. Morandi, Neta Regev-Rudzki

**Affiliations:** † Department of Biomolecular Sciences, Faculty of Biochemistry, Weizmann Institute of Science, Rehovot 7610001, Israel; ‡ Department of Chemical Research Support, Weizmann Institute of Science, Rehovot 7610001, Israel; § Bioinformatics Unit, Life Sciences Core Facilities, Weizmann Institute of Science, Rehovot 7610001, Israel; ∥ Raymond and Beverly Sackler Faculty of Exact Sciences, School of Chemistry, Tel Aviv University, Tel Aviv 6997801, Israel; ⊥ Flow cytometry Unit, Life Sciences Core Facilities, Weizmann Institute of Science, Rehovot 7610001, Israel; # Institute of Organic Chemistry and Biochemistry of the Czech Academy of Science, Prague 160-00, Czech Republic; ∇ IMol Polish Academy of Sciences, Warsaw 02-247, Poland

**Keywords:** extracellular vesicles, EVs, malaria, cellular uptake, membrane deformability, imaging flow cytometry

## Abstract

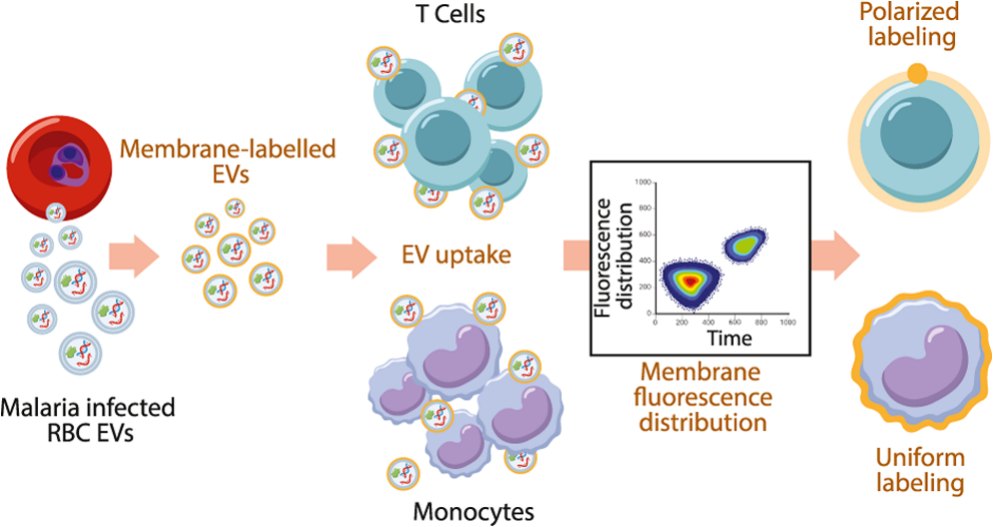

The malaria parasite, *Plasmodium falciparum*, secretes extracellular vesicles (EVs) to facilitate its growth and to communicate with the external microenvironment, primarily targeting the host’s immune cells. How parasitic EVs enter specific immune cell types within the highly heterogeneous pool of immune cells remains largely unknown. Using a combination of imaging flow cytometry and advanced fluorescence analysis, we demonstrated that the route of uptake of parasite-derived EVs differs markedly between host T cells and monocytes. T cells, which are components of the adaptive immune system, internalize parasite-derived EVs mainly through an interaction with the plasma membrane, whereas monocytes, which function in the innate immune system, take up these EVs via endocytosis. The membranal/endocytic balance of EV internalization is driven mostly by the amount of endocytic incorporation. Integrating atomic force microscopy with fluorescence data analysis revealed that internalization depends on the biophysical properties of the cell membrane rather than solely on molecular interactions. In support of this, altering the cholesterol content in the cell membrane tilted the balance in favor of one uptake route over another. Our results provide mechanistic insights into how *P. falciparum*-derived EVs enter into diverse host cells. This study highlights the sophisticated cell-communication tactics used by the malaria parasite.

Malaria remains one of the most devastating infectious diseases worldwide, responsible for more than 600 000 deaths in 2022.^1^ The disease is caused by protozoan parasites of the genus *Plasmodium*, with most of the mortality being attributed to *Plasmodium falciparum*. Pathogens utilize several modes of cell communication, including the evolutionary conserved release of extracellular vesicles (EVs).^2,3^ EVs mediate intercellular communication by delivering a wide range of bioactive components, including proteins and nucleic acids, from one cell to another.^4,5^ These vesicles are released from cells in rest and upon induction in order to induce significant phenotypic changes in the recipient target cells.^4^ EVs are composed of a lipid bilayer, display extracellular plasma membrane features on their surfaces,^6−8^ and transport cell type-specific cargoes that are important for their functions.^9−11^

From inside their host red blood cells (RBCs), *P. falciparum* parasites secrete EVs^12^ that alter host responses.^13−15^ Asymmetric flow field-flow fractionation of *P. falciparum*-derived EVs revealed two distinct EV subpopulations differing in size and protein content.^16^ A detailed characterization of *P. falciparum*-derived EV composition revealed various functional biomolecules, including enrichment of multiple host and parasitic proteins,^16−18^ particularly parasite antigens and proteins associated with the host cell membrane.^13,16,18,19^ The vesicles also contain parasitic genomic DNA^5^ and RNA,^5,14,20^ which are involved in immunogenic responses in human recipient cells.^14,17,21^

Various mechanisms for EV uptake have been suggested, including clathrin-mediated endocytosis, phagocytosis, macropinocytosis, and plasma or endosomal membrane fusion.^22−26^ Different subsets of EVs may utilize different routes to enter a target cell.^26,27^ Although EVs are taken into the endosomal compartment by endocytosis,^22,24,27−32^ the precise mechanism regulating the internalization of EVs is a subject of considerable debate. Furthermore, lipid raft proteins and particular protein–protein interactions appear to be important in the uptake of EVs.^24^ EV attachment and subsequent internalization may be aided by protein–protein interactions involving membrane receptors, ligands, or adhesive proteins on recipient cells such as tetraspanins, lectins, proteoglycans, and integrins.^24^

Furthermore, it is suggested that EV uptake is an extremely rapid process, with EV cargo detected inside target cells within minutes after initial introduction to the cells.^33,34^ Thus, the current evidence suggests that the uptake of EVs is a rapid and energy-dependent process that relies on a functioning cytoskeleton, indicating the involvement of endocytic pathways. A few studies have suggested that direct fusion between EVs and the plasma membrane of the recipient cells could be an alternative route for EV internalization.^28,35^ For example, it was shown that acidic microenvironments increased the entry of tumor-derived exosomes into melanoma cells, mediated by a lipid-dependent fusion process, which is resistant to paraformaldehyde fixation.^35^ Thus, it seems that multiple mechanisms, likely dependent on EV characteristics and the host cell type, lead to EV internalization.^26^ Most research has focused on the uptake of human-derived EVs by target cells, and therefore, our understanding of how cells take up EVs derived from malaria and other parasites remains limited.^36^ One major challenge in the determination of the exact uptake route of EVs, especially malaria-derived EVs, is the lack of biological markers of EVs.^37,38^

As diverse processes involving cellular membranes are influenced by the membrane’s physical properties,^39,40^ as demonstrated in cases of viral entry^41^ and drug delivery,^42^ we investigated the biophysical mechanisms underlying the uptake of *P. falciparum*-derived EVs. Specifically, we aimed to determine whether there are significant differences in the uptake routes between host cells of the innate and adaptive immune systems, human monocytes (THP-1 cells) versus T cells (Jurkat cells), respectively. Using imaging flow cytometry (IFC) analysis of labeled EVs, we identified two distinct patterns in the different recipient cells. A membranal fluorescence “capped” (or “hot spot” type) signal appeared on the T cell membrane, whereas labeling was uniformly distributed throughout the cellular membrane of monocytes, likely due to endocytosis. The mechanical deformability and stiffness of the host cell membrane were crucial in determining the entry path of the *P. falciparum-*derived EVs: T cells have stiffer membranes than monocytes, and depletion of cholesterol from the T cell membrane, which makes it less rigid, resulted in a switch to internalization via endocytosis. This study provides a biophysical framework to understand the internalization of malaria-derived EVs into target cells and provides insights into how the malaria parasite targets specific subsets of the immune system to its benefit, opening the door to identifying potential intervention targets to limit malaria virulence.

## Results and Discussion

### Analysis of Signal Distribution in Live Cells During EV Uptake Using a Self-Quenching Lipid Dye

In order to characterize the uptake dynamics of *P. falciparum*-derived EVs in two types of host immune cells, monocytes and T cells, we implemented an IFC method that had been employed previously to analyze the distribution of the RNA cargo of *P. falciparum*-derived EVs.^38,43^ The advantage of IFC is that it allows for robust screening of over 100 000 cells within a short time,^44^ enabling high-throughput analysis while also providing the capability to characterize individual cell features. This facilitates the identification of subpopulations within the heterogeneous pool of cells. To specifically focus on dissecting the EV entry route into host immune cells, we exploited the fluorescence properties of the lipophilic self-quenching dye octadecyl rhodamine B (R18).^45−47^ At high concentrations within a lipid bilayer, the fluorescence of R18 is suppressed due to self-quenching; upon dilution, either due to membrane disruption or membrane fusion (e.g., when the EV labeled lipids are incorporated in the recipient cell membrane), R18 fluorescence intensity sharply increases.^46,47^ This property has been extensively exploited to measure membrane fusion in viruses and other systems.^28^

**Figure 1:**
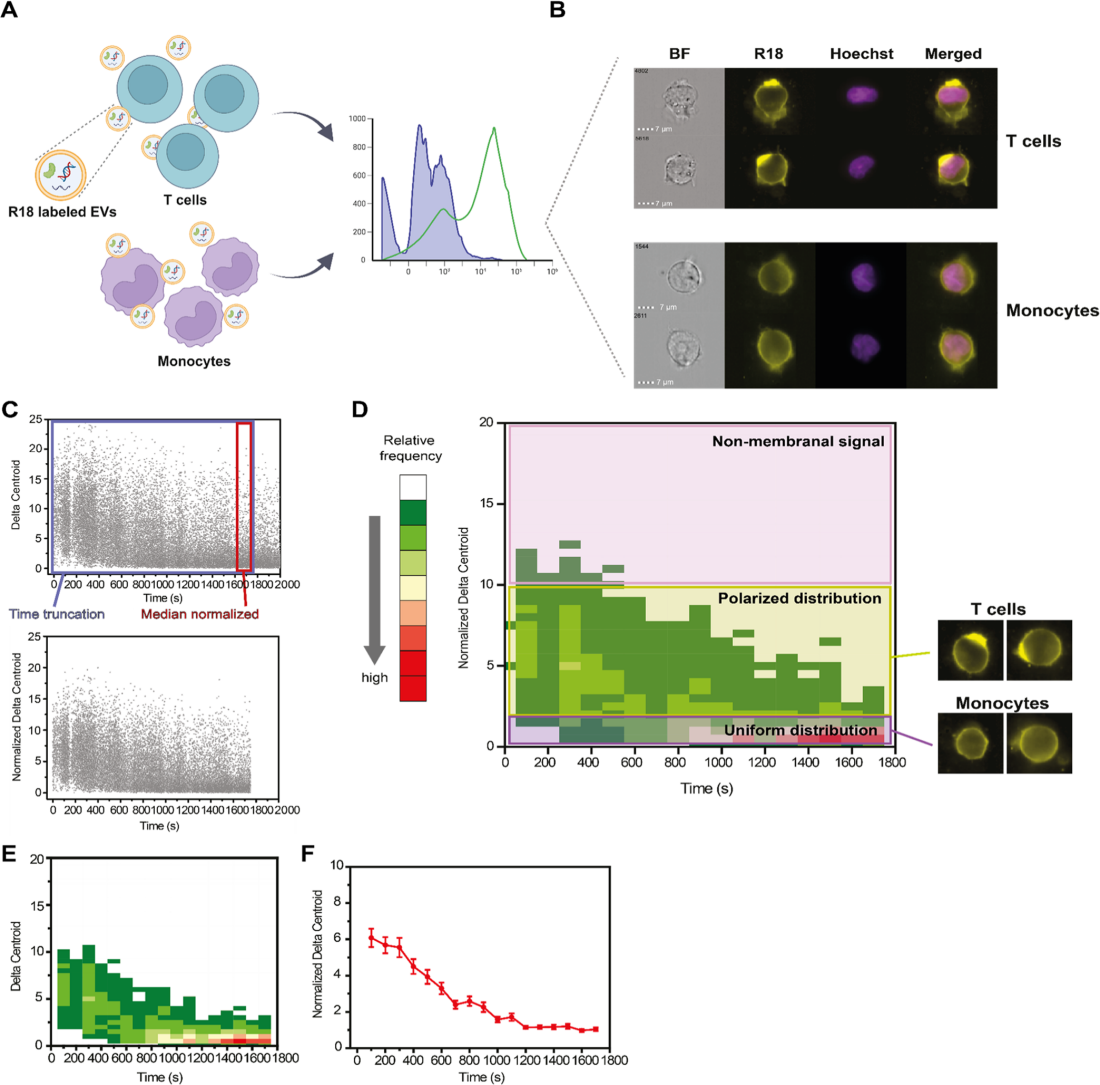
Analysis of fluorescence signal from R18-labeled EVs reveals differences in uptake patterns into T cells and monocytes. (A) Graphical illustration of the experimental setup utilized to probe *P. falciparum*-derived EV uptake by cells. R18-labeled EVs were incubated with Hoechst-labeled cells within the flow chamber of the IFC instrument, and fluorescence images were continuously acquired throughout the 30 min incubation. (B) Representative IFC images acquired in the bright field (general shape and morphology of the cell), in the R18 channel (yellow), and in the Hoechst channel (purple) for the nuclear signal to select live cells. (C) Representative data distribution of the Δ_*xy*_ parameter obtained from an acquisition run. Data was then time-consumed and median normalized for easier comparison to other acquisitions. (D) Heat map obtained from 2D binning the distribution of normalized and truncated data, displaying relative frequencies of events in a specific time and Δ*xy* coordinates. Representative images of the R18 channels for cells with polarized and uniform distributions (T cells and monocytes, respectively) are shown. (E) Filtered frequency distribution obtained after removing all values below statistical noise from the data plotted in panel D. (F) Kinetic profile obtained by using relative frequencies from panel E to calculate the weighted average for Δ_*xy*_^norm^. Data are presented as weighted averages (dots) with weighted standard errors (whiskers). Illustrations created with BioRender.com and licensed for publication (agreement number: VK26WKI424).

We isolated EVs from *P. falciparum-*infected RBCs, characterized the EVs (Figure S1), and labeled their membranes using R18. After validating the incorporation of the R18 dye into the isolated EVs (Figure S2), we introduced the EVs into cultures of either human monocytes (THP-1 cells) or human T cells (Jurkat cells). The fluorescence signal of the R18-labeled EVs was measured using IFC for 30 min in a live acquisition manner^5,38^ (Figure 1A,B). Subsequently, we truncated the data sets to the shortest common time point to compare different acquisitions. We normalized each curve by its respective median obtained from all data points in the last truncated 100 s (Figure 1C).

To characterize the EV uptake signal pattern in each host cell type, we focused on two primary parameters of the obtained images: the total fluorescence intensity and the fluorescence spatial distribution in the membrane of the recipient host cells. For each image, the fluorescence spatial distribution was quantified based on the Δ_*xy*_ feature.^48^ This feature measures the distance between the geometrical fluorescence centers for the images in the two separate channels.^44^ In our case, Δ*_xy_* was calculated as the distance (in μm) from the center of the bright-field image (which is located at the geometrical center of the cell) to the center of the intensity-weighted R18 labeling. In the case of uniformly distributed labeling throughout the cellular membrane (meaning the center of the labeling is at the geometrical center of the cell), the distance was small and constant (Figure 1D). In contrast, a high Δ*_xy_* value is indicative of a longer distance between the center of mass of the fluorescence and the geometrical center of the cell,^44^ meaning that the dye occupies a polarized localization within the cell or cell membrane (Figure 1D). Each data set was then 2D-binned both in time (100 s bin width) and based on normalized total fluorescence intensity (0.1 bin width) and based on normalized Δ*_xy_* (Δ_*xy*_^norm^; 0.5 bin width), and the relative frequency for each bin was calculated. With this approach, we can visualize the differences in the uptake behavior between the two cell types (Figure 1D).

Comparison of the Δ_*xy*_^norm^ frequency distribution for each cell type incubated with R18 dye without EVs showed that both the pattern observed and differences between cells were not due to different dye internalization but rather the interaction with EVs (Figure S3). Three patterns were observed (Figure 1D): (i) A uniform membrane distribution region (0 < Δ_*xy*_^norm^ < 2), predominantly observed in monocytes. (ii) A polarized membranal signal region, either in the form of a fluorescence cap or puncta (2 < Δ_*xy*_^norm^ < 10), which appeared primarily in T cells at early time points. (iii) We also observed fluorescence puncta not associated with the cell membrane region (Δ_*xy*_^norm^ > 10); this pattern was observed in both cell types and likely does not have biological relevance. In addition, using the obtained relative frequencies as a weight for statistics and filtering out frequencies below statistical randomness (Figure 1E), we extracted the kinetic profiles of each parameter to infer additional information on the EV uptake process (Figure 1F).

### Total and Membrane-Distributed Signals Differ in Recipient Monocytes and T Cells

**Figure 2:**
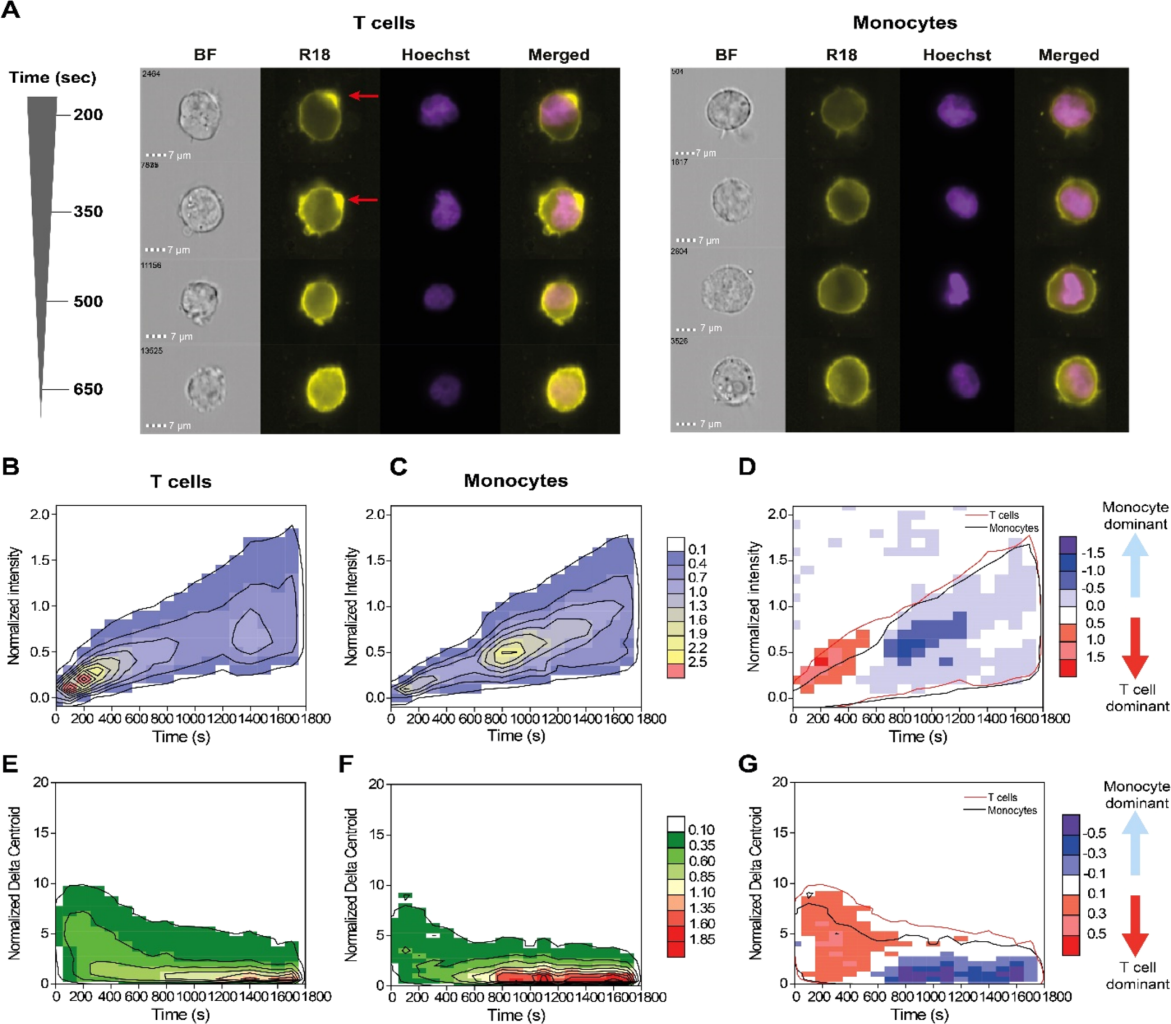
Total intensities and intensity distributions of R18-labeled EVs are different between T cells and monocytes. (A) Representative IFC images of recipient T cells (left) and monocytes (right) showing the differences in the R18 signal distributions over time during incubation with R18-labeled *P. falciparum*-derived EVs. (B, C) Average heat maps of total R18 fluorescence intensity (i.e., amount of fluorescence measured in the whole cell) after incubation of R18-labeled *P. falciparum*-derived EVs with either (B) T cells or (C) monocytes. Heat map color represents the relative frequency (in percentage) at each specific normalized intensity - time coordinate, according to color legend in the figure. (D) Heat map of R18 total intensity events in T cells and monocytes over time. Blue areas indicate more events in monocytes compared with T cells, and red areas indicate the opposite. Outlines indicate the isofrequency region at a relative frequency of 0.1% for either T cells (red) or monocytes (black). Heat map color represents the difference in relative frequency (in percentage) between T cells and monocytes legend in the figure. (E, F) Average heat maps of cellular R18 Δ_*xy*_^norm^, representative of uniformity of fluorescence signal along the membrane, after incubation of R18-labeled *P. falciparum*-derived EVs with either (E) T cells or (F) monocytes. Heat map color represents the relative frequency (in percentage) at each specific Δ_*xy*_ - time coordinate, according to color legend in the figure. (G) Heat map of R18 Δ_*xy*_^norm^ events in T cells and monocytes. Blue areas indicate more events in monocytes compared to T cells, and red areas indicate the opposite. Outlines indicate the isofrequency region at a relative frequency of 0.1% for either T cells (red) or monocytes (black). Heat map color represents the difference in relative frequency (in percentage) between T cells and monocytes at each specific Δ_*xy*_^norm^ - time coordinates, according to color legend in the figure. All panels were obtained from 5 independent biological repeats.

Analysis of the R18 fluorescence signals revealed apparent differences in the total intensities and Δ*_xy_* in T cells and monocytes post-uptake of *P. falciparum*-derived EVs. The total R18 intensity increased steadily over time in both cell types (Figures 2A–C and S4A,B), with T cells showing a higher event frequency at low normalized intensity values (∼0.25) in the first 400 s time window than monocytes (Figures 2B and S4A). The intensity distribution in monocytes was more homogeneous over time, with only some clustering in the ∼800 s region (Figures 2C and S4B). The difference in frequencies between the two cell types confirms these distinct behaviors, with an early high signal observed on the membranes of T cells but not on the membranes of monocytes, consistent with the polarized signal observed on the T cell membrane (Figure 2D). Monocytes have low and constant Δ_*xy*_ values across all time points, suggesting a uniform distribution of the signal along the cell membrane through the entire uptake process, whereas, in T cells, we observed an accumulation of the lipid dye at a certain region of the membrane immediately upon EV introduction (Figures 2A,E–G and S4D). Indeed, T cells have significantly more Δ_*xy*_^norm^ events in the polarized region (2 < Δ_*xy*_^norm^ < 10) at lower time points (<500 s) than observed in monocytes (Figures 2D,E–G and S4C). The membranal capped signal in T cells gradually decreased over time to more uniform values (Δ_*xy*_^norm^ < 2, Figure 2E). Moreover, monocytes have lower Δ*_xy_* values at later time points (>1000 min) than do T cells (Figures 2F and S4D). Analysis of the difference in distributions between the two cell types show a propensity for T cells to have highly polarized cells early on, and monocytes have a consistent distribution of fluorescence signals surrounding their cell membrane, which gradually accumulates (Figure 2G). In both cell types, the EV cargo is fully internalized, as labeling of EVs with TO, which binds to RNA, and subsequent incubation with either monocytes^28^ or T cells (Figure S5) result in the intracellular TO fluorescence signal that increases over time.

**Figure 3:**
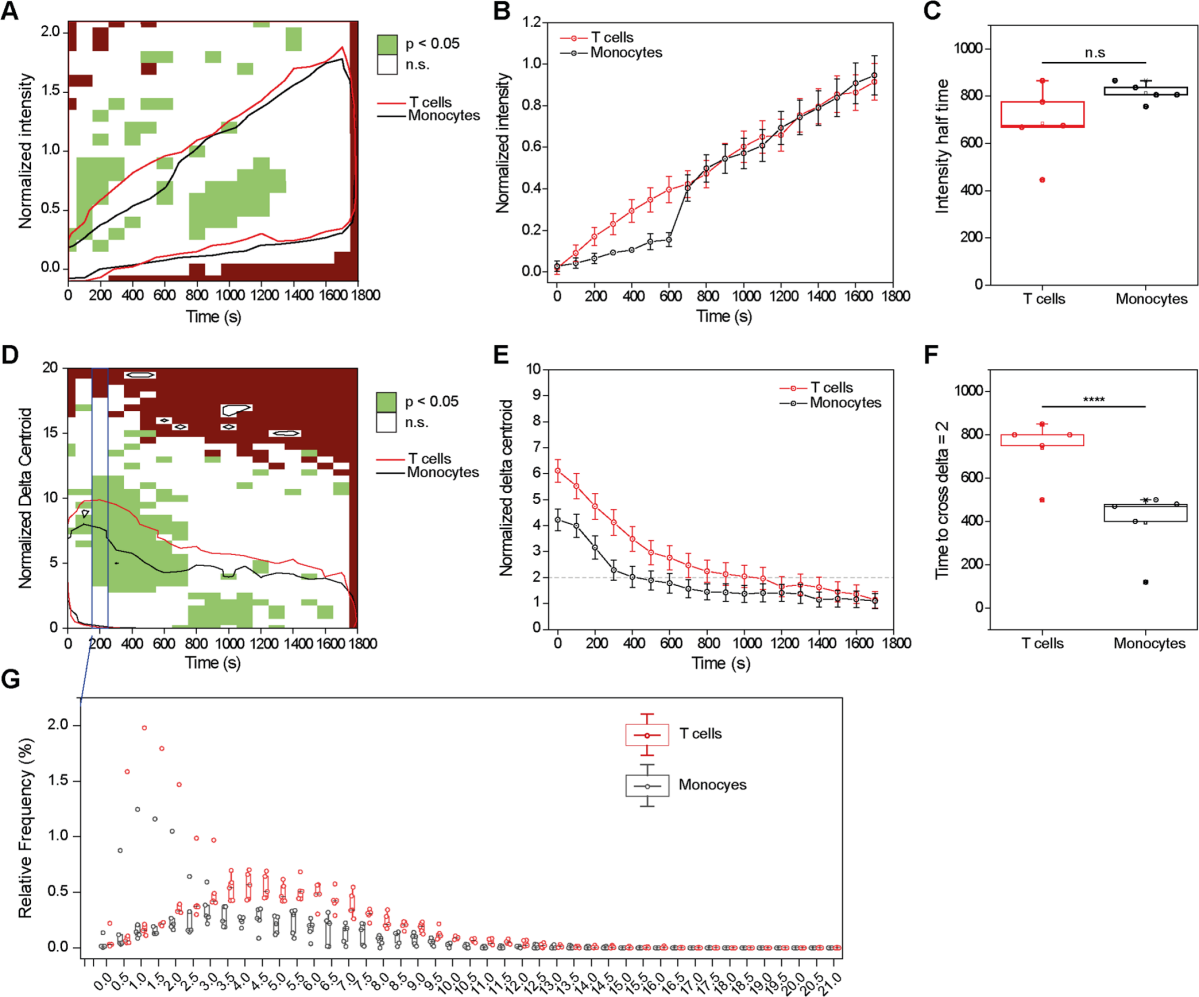
Fluorescence intensity distribution reveals differential uptake mechanisms in T cells versus monocytes. (A) Heat map of statistical significance of total R18 fluorescence intensity postincubation of R18-labeled EVs into T cells and monocytes obtained from IFC images. Statistical significance was calculated using a two-sample *t*-test when green indicates statistical significance (*p* < 0.05), white indicates no significance (n.s.), and brown indicates statistical test not applicable. Outlines indicate the isofrequency region at a relative frequency of 0.1% for T cells (red) and monocytes (black). (B) Representative kinetic profiles of total R18 fluorescence intensity in T cells (red) and monocytes (black). (C) Comparison of τ^1/2^ values for T cells and monocytes, showing no significant difference (n.s.). (D) Heat map of statistical significance in R18 fluorescence Δ_*xy*_^norm^ after internalization of R18-labeled EVs to T cells and monocytes obtained from IFC images. Statistical significance was calculated using a two-sample *t*-test when green indicates statistical significance (*p* < 0.05), white indicates no significance (n.s.), and brown indicates statistical test not applicable. Outlines indicate the isofrequency region at a relative frequency of 0.1% for T cells (red) and monocytes (black). (E) Representative kinetic profiles of total R18 fluorescence Δ*_xy_* in T cells (red) and monocytes (black) throughout the time window. (F) Comparison of time to reach homogeneous fluorescence distribution (time to cross Δ_*xy*=2_^norm^) for T cells and monocytes. (G) 2D distribution of the relative frequency of events binned across Δ*_xy_* values (bin width of 0.5) and time (bin width of 100 min) for T cells (red) and monocytes (black).

Statistical analysis of the total R18 intensity revealed actual differences between T cells and monocytes only in the 800–1200 s region, with some sporadic statistical significance in areas where the main kinetics does not occur (Figure 3A). The frequency-weighted kinetic profiles indicate differences in the time evolution of the total intensity, primarily during the early stages of EV uptake. Monocytes have a slower increase from 0 to 600 s followed by complete overlap with T cell kinetics (Figures 3B and S6A). These differences, however, are not enough to fully differentiate the two cell types. For instance, the estimation of the half-time (τ^1/2^, the time point at which the total intensity is half of the final value) for each cell type revealed no significant difference (Figure 3C) with a τ^1/2^ of 686 ± 157 s for T cells and 814 ± 41 s for monocytes (weighted average ± weighted standard error, *n* = 5, *p* = 0.12). In contrast, statistical analysis of Δ_*xy*_^norm^ showed a significant (*p* < 0.05) difference in the Δ*_xy_* values at early time points (<500 s) and higher Δ*_xy_* values (5–10 Δ*_xy_*) as well as in the low Δ*_xy_* (0–2.5 Δ*_xy_* region) at medium and later times (750–1600 s, Figure 3D). This is in line with the major differences observed in the two frequency distributions (Figure 2G).

Further, the kinetic profiles are distinctly different between the two cell types, with T cells having slower kinetics (Figures 3E and S6B). Unlike the profile of T cells, the kinetic profile of the monocytes rapidly decays into a homogeneous Δ_*xy*_^norm^ region and stabilizes. Estimation of the time to reach homogeneous distribution upon EV uptake (τ^Δ=2^, time at which Δ_*xy*_^norm^ = 2) revealed that, indeed, two cell types have distinct and significantly different behaviors with τ^Δ=2^ of 804 ± 131 s for T cells and 425 ± 241 s for monocytes (weighted average ± weighted standard error, *n* = 5, *p* = 0.015; Figure 3F). These differences were further highlighted by investigation of individual distributions for each time bin: more statistically significant differences were observed in the region at 3–5 Δ_*xy*_^norm^ values (Figure 3G).

Visualization using fluorescence confocal microscopy of the two cell types after a 10 min incubation with R18-labeled EVs confirmed the flow cytometry observations (Figure S7). The membranal R18 fluorescence intensity in T cells was significantly higher than that in monocytes, in agreement with the extrapolated kinetic curves at 10 min (Figure 3B). For T cells, higher-resolution confocal microscopy confirmed the presence of the capping morphology observed by IFC, with segments of the cell membrane displaying enhanced intensity (Figure S7A,B). In contrast, in monocytes, the fluorescence intensity was much more uniform (Figure S7A,B). Estimation of the occurrence of capping was consistent between the imaging techniques employed with T cells about twice as likely as monocytes to display a polarized membrane fluorescence (Figure S7C). Interestingly, although monocytes had a lower fluorescence intensity at the plasma membrane than T cells, we observed a large amount of high-intensity intracellular fluorescence puncta in close proximity to the cell membrane in monocytes (Figure S7D). This feature is not observed as prominently in T cells. In addition, we observe that the number of fluorescence puncta in the bulk, which we interpret as EVs in solution, was much higher in T cells compared to monocytes (Figure S7E).

Together, these results suggest significant differences in the *P. falciparum*-derived EV uptake mechanisms in the two types of immune cells. Monocytes are highly phagocytic cells;^49,50^ thus, they might internalize the parasitic EVs mainly by the endocytosis pathway, which is a rapid and highly regulated process.^51−54^ In support of this hypothesis, we previously demonstrated that the internalization of *P. falciparum*-derived EV cargo into these host cells occurs rapidly within a matter of a few minutes.^43^ Confocal images of monocytes after a 10 min incubation with R18-labeled EVs showed distinct bright intracellular puncta (likely endosomes) and fewer EVs in bulk in monocytes than in T cells, suggestive of rapid EV entry into monocytes. The R18 lipid labeling distribution is uniform throughout the uptake process into monocytes, but the signal is initially of low intensity as only part of R18 has been mixed with the plasma membrane, resulting in enough dilution to result in loss of self-quenching. Upon full recycling of the membrane following endocytosis, a process that occurs in 10–20 min,^43^ the signal remains uniform but increases in intensity. In the case of the T cells, however, the internalization of EVs arises primarily from direct interaction with the plasma membrane, which may account for the capping effect that we observe early during the uptake. This direct EV membrane fusion mechanism is not yet completely understood, unlike EV internalization via endocytosis, which has been extensively detailed.^55^

### EV Uptake Kinetics Differ in Monocytes versus T Cells, as Shown by Spectral Flow Cytometry

As a complementary approach to IFC, we employed spectral flow cytometry to examine the differences in the uptake kinetics of *P. falciparum*-derived EVs between recipient monocytes and T cells. We either labeled the EV membrane using R18 or the RNA cargo of the EVs using TO dye^5,38,43^ and introduced the *P. falciparum*-derived EVs to either monocytes or T cells for 5, 15, and 30 min. Upon incubation with R18-labeled EVs, there was a clear difference in the uptake kinetics between the two immune cell types, with T cells showing faster kinetics in the initial 5 min compared with the monocytes (Figure S8). This result is in agreement with the observations from the IFC experiments, in which we saw a higher event frequency at low normalized intensity values in the T cells compared to a more homogeneous distribution in the monocytes in the first 400 s time window (Figures 3B and S6A).

**Figure 4:**
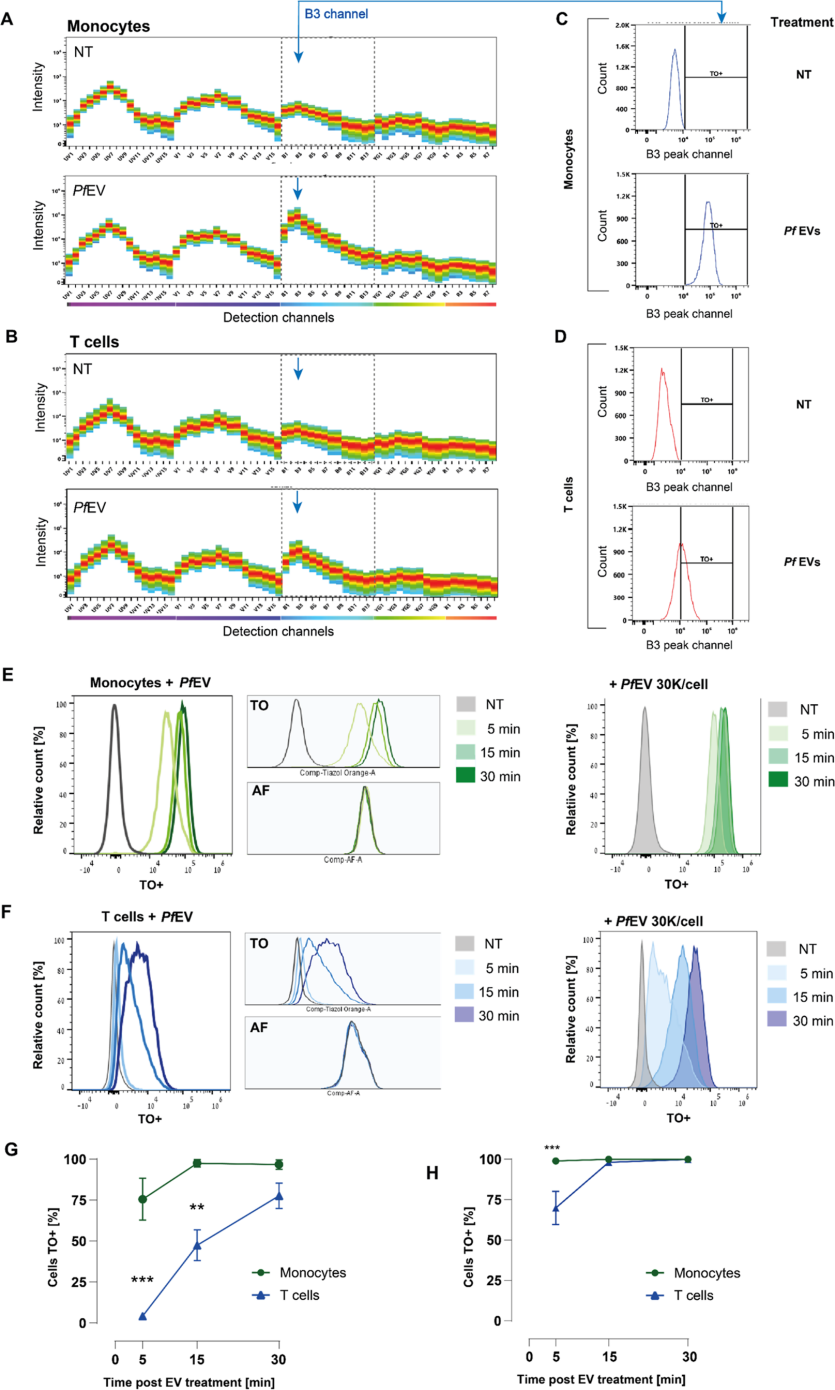
Monocytes and T cells have distinct *P. falciparum*-derived EV uptake kinetics. (A, B) Representative images of spectral signatures of (A) monocytes and (B) T cells either untreated or treated for 30 min with TO-labeled *P. falciparum*-derived EVs. The areas within gray dashed lines show B1–B14 channels equivalent to the TO fluorescence signal range. (C, D) Representative pseudocolor (left) and histogram (right) plots for raw B3 peak channel intensities in TO-positive (C) monocytes and (D) T cells. (E, F) Representative post-unmixing histograms (with autofluorescence subtraction) illustrating time-dependent increases in fluorescence signal following 5-, 15-, or 30 min incubation with TO-labeled *P. falciparum*-derived EVs in (E) monocytes and (F) T cells. The adjacent histograms show treatment-induced changes (upper) versus autofluorescence levels (lower) across experimental groups. (G, H) Averaged percentages of TO^+^ cells for (G) 10 000 EVs per cell with statistical significance evaluated using ANOVA *F*(5, 16) = 22.7, *p* < 0.0001 and (H) 30 000 EVs per cell with statistical significance evaluated using ANOVA *F*(5, 16) = 7.1, *p* < 0.01, Šidak multiple comparison tests. **p < 0.01, ***p < 0.001 monocytes versus T cells within the same time points.

When focusing on the EV cargo, the initial analysis of monocytes and T cells exposed to TO-labeled EVs revealed a positive signal across all emissiondetection channels excited by blue laser (498–824 nm) with a higher signal in monocytes than T cells (Figure 4A,B). The signal corresponded to the TO dye known excitation/emission spectrum (513/533 nm). These data suggest that the uptake of EVs by monocytes is more efficient than the uptake by T cells. We next compared the experimental samples using the raw fluorescence intensity values obtained within the B3 channel (533–550 nm), the peak emission channel in EV-treated monocytes and T cells (Figure 4C,D). We found that after 5 min of incubation with *P. falciparum*-derived EVs, most monocytes (84.1 ± 9.3%) were positive for TO, whereas significantly fewer T cells (4.9 ± 2.3%) were TO positive at the same time point (Figure S9). The disparity in the uptake pattern was sustained at 15 and 30 min post-EV introduction. These two distinct uptake patterns per cell type remained significant even at a higher EV concentration (98.9% for monocytes and 56.5% for T cells after 5 min; Figure S9).

Immune cells may exhibit autofluorescence,^56^ raising the probability of interference with the fluorescence assays.^57^ To test whether the observed effects stem from changes in intrinsic cell autofluorescence, we applied an unmixing algorithm to the detected fluorescence cell profiles to reveal cell line-specific autofluorescence signatures. This analysis confirmed that monocyte EV uptake is significantly faster than uptake into T cells across all time points and EV concentrations tested with no changes in the background autofluorescence (Figure 4E,F). Specifically, most monocytes (75%) were TO positive after 5 min of EV incubation, whereas only about 4% of the T cells were TO positive (Figure 4G). This significant difference in the uptake efficiency was also apparent at a higher EV concentration (Figure 4H).

### Removal of Intracellular Organelles Eliminates the Uptake Differences between Monocytes and T Cells

**Figure 5:**
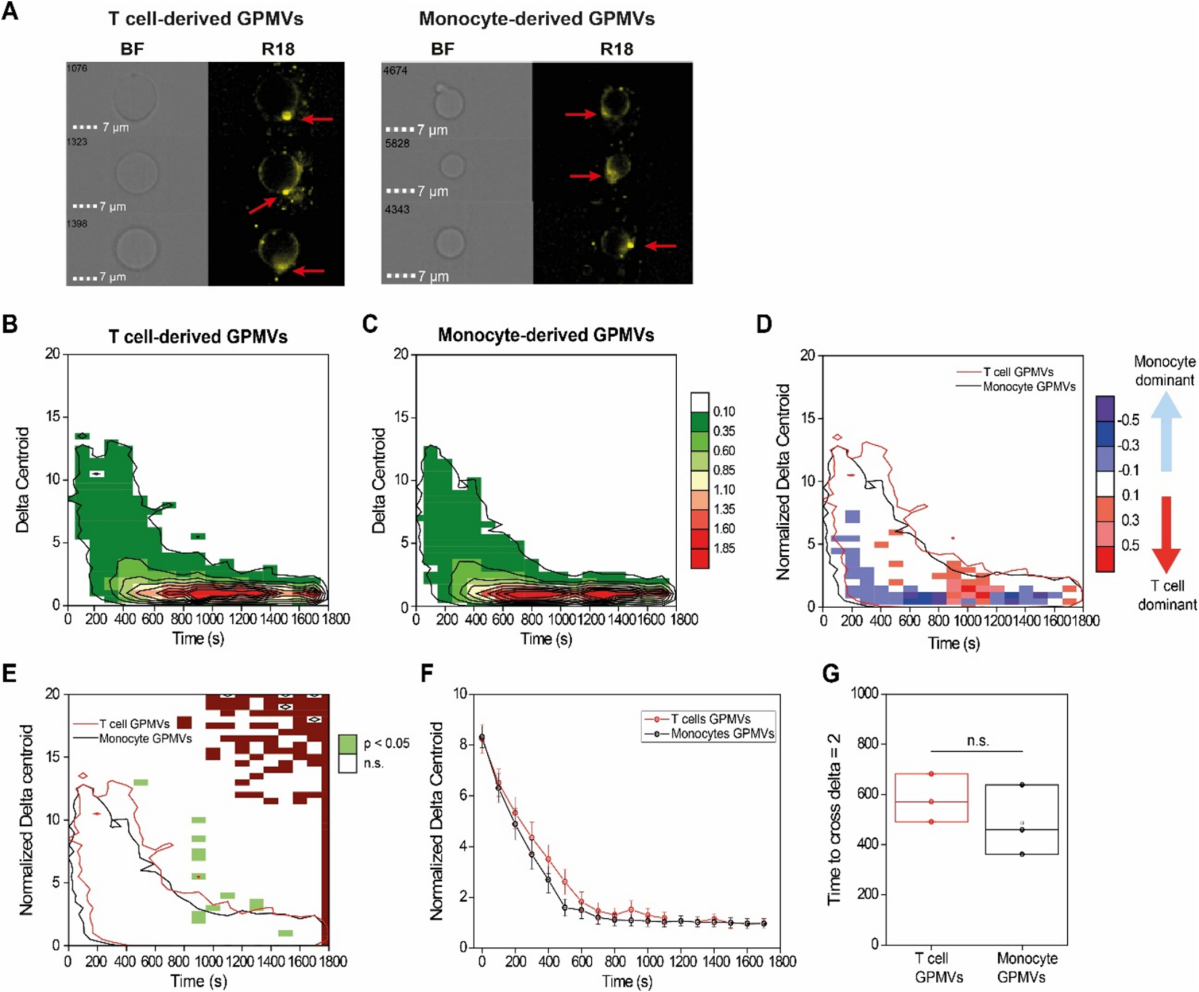
Depletion of cellular organelles removes differences in EV internalization dynamics between T cells and monocytes. (A) Representative images of T cell-derived (left) and monocyte-derived (right) GPMVs following incubation with R18-labeled EVs. Images are shown in the bright field (BF) and the R18 fluorescence channel. (B) Heat map of the average R18 Δ*_xy_* relative frequency after incubation of R18-labeled EVs with T cell-derived GPMVs. (C) Heat map of average R18 Δ_*xy*_ relative frequency after incubation of R18-labeled EVs with monocyte-derived GPMVs. (D) Heat map of R18 Δ_*xy*_^norm^ events in T cells and monocytes. Blue areas indicate more events in monocytes compared to T cells, and the red areas indicate the opposite. Outlines indicate the isofrequency region at a relative frequency of 0.1% for T cells (red) and monocytes (black). (E) Heat map of statistical significance in R18 fluorescence Δ_*xy*_^norm^ after internalization of R18-labeled EVs in GPMVs derived from T cells and monocytes. Significance was determined by using a two-sample *t*-test with green indicating statistical significance (*p* < 0.05), white indicating no significance (n.s.), and brown indicating statistical test not applicable. (F) Representative total R18 fluorescence Δ*_xy_* profiles for GPMVs derived from T cells (red) and monocytes (black). (G) Comparison of time to reach homogeneous fluorescence distribution (time to cross Δ_*xy*_^norm^ = 2) for GPMVs derived from T cells and monocytes. The data presented are based on 3 biological repeats. Each kinetic profile is presented as a weighted average (dot) and weighted standard error (whiskers). Heat map color in panels B and C represents the relative frequency (in percentage) at each specific Δ_*xy*_ - time coordinate, according to color legend in the figure. Heat map color in panel D represents the difference in relative frequency (in percentage) between T cells and monocytes at each specific Δ_*xy*_^norm^ - time coordinates, according to color legend in the figure.

Next, we investigated whether the plasma membrane characteristics of the host immune cells determine the routes of uptake (capped versus uniform for T cells and monocytes, respectively). For that, we segregated the plasma membrane of each cell type by generating giant plasma membrane vesicles (GPMVs) from T cells and monocytes.^28^ GPMVs are commonly used for efficiently isolating intact plasma membranes (PMs).^48^ The GPMVs are filled with cytoplasm but free from internal organelles and maintain the native membrane compositions of proteins and lipids.^48,71^ We first validated that the GPMVs have spherical morphology by using atomic force microscopy (AFM) (Figure S10) and that they do not contain any internal organelle components such as DNA (Figure S11). We then mixed GPMVs derived from T cells and monocytes with R18-labeled *P. falciparum-*derived EVs and measured the acquired fluorescence signal using IFC, as was previously done for intact cells. In this case, we monitored the distribution of the EV signal interacting solely with the plasma membranes. We found that the Δ_*xy*_ distributions over time were comparable between GPMVs derived from T cells and monocytes (Figure 5A–C), with the capped characteristic distribution in the polarized Δ_*xy*_^norm^ at early times, typical of live T cells, observed for both GPMV types. Comparison of the two GPMV distributions shows minor differences, with monocyte GMPVs displaying more events in the low delta values across all time points and T cell GMPVs showing higher frequency in the 1–4 Δ_*xy*_^norm^ region at ∼800 s (Figures 5B–D, S12, and S13).

No difference was detected in the Δ_*xy*_ distributions between monocyte- and T cell-derived GPMVs (Figure 5B,C). Further analysis of the kinetic profiles of the two GPMV types revealed almost total overlap between the two populations (Figures 5D–F and S14). No difference was detected in the time required to reach a homogeneous distribution of the R18 signal with τ^Δ=2^ of 566 ± 115 s for T cells and 473 ± 145 s for monocytes (Figure 5G). Although removal of the intracellular organelles minimally changed the Δ_*xy*_^norm^ pattern and kinetic profiles for T cells, it drastically modified it for monocytes with a clear increase and statistical significance of events in the capped Δ_*xy*_^norm^ region at early times (100–600 s) and reduction in the uniform region at later times (>800 s) (Figure S13). The observed effects are not byproducts of the GPMV production reagent on the membrane, as treatment of both cells with DTT and subsequent evaluation of the resulting Δ_*xy*_^norm^ profiles showed an opposite trend to GPMV uptake, with cells becoming more endocytic (Figure S15).

In the experimental setup with GPMVs, no active or energy-dependent processes take place. Thus, these results suggest that the membranes of T cells and monocytes have similar affinities for EVs. The entry of EVs into GPMVs from both cell types is comparable, with no significant dynamic differences. Since the removal of intracellular organelles eliminated the typical uniform pattern of low Δ_*xy*_^norm^ at early times exhibited by monocytes and reverted it into a T cell-like capped pattern, these data support our notion that monocyte internalization of *P. falciparum*-derived EVs probably occurs primarily via endocytosis.

### AFM Analysis Reveals that the T Cell Membrane is Stiffer than the Monocyte Membrane

Having demonstrated that the major difference in EV internalization by T cells and monocytes is the balance between endocytosis and direct interaction at the plasma membrane, we sought to investigate whether there are differences in the properties of the host cell membranes that contribute to such a balance. Endocytic pathways, such as clathrin-mediated or caveolin-mediated endocytosis,^58^ are modulated by distinct protein machinery, but the mechanism of endocytosis, regardless of type, is bound by the physical parameters of the cell membrane.^40^ Transitioning from the plasma membrane to inward budding requires mechanical force.^40^

**Figure 6:**
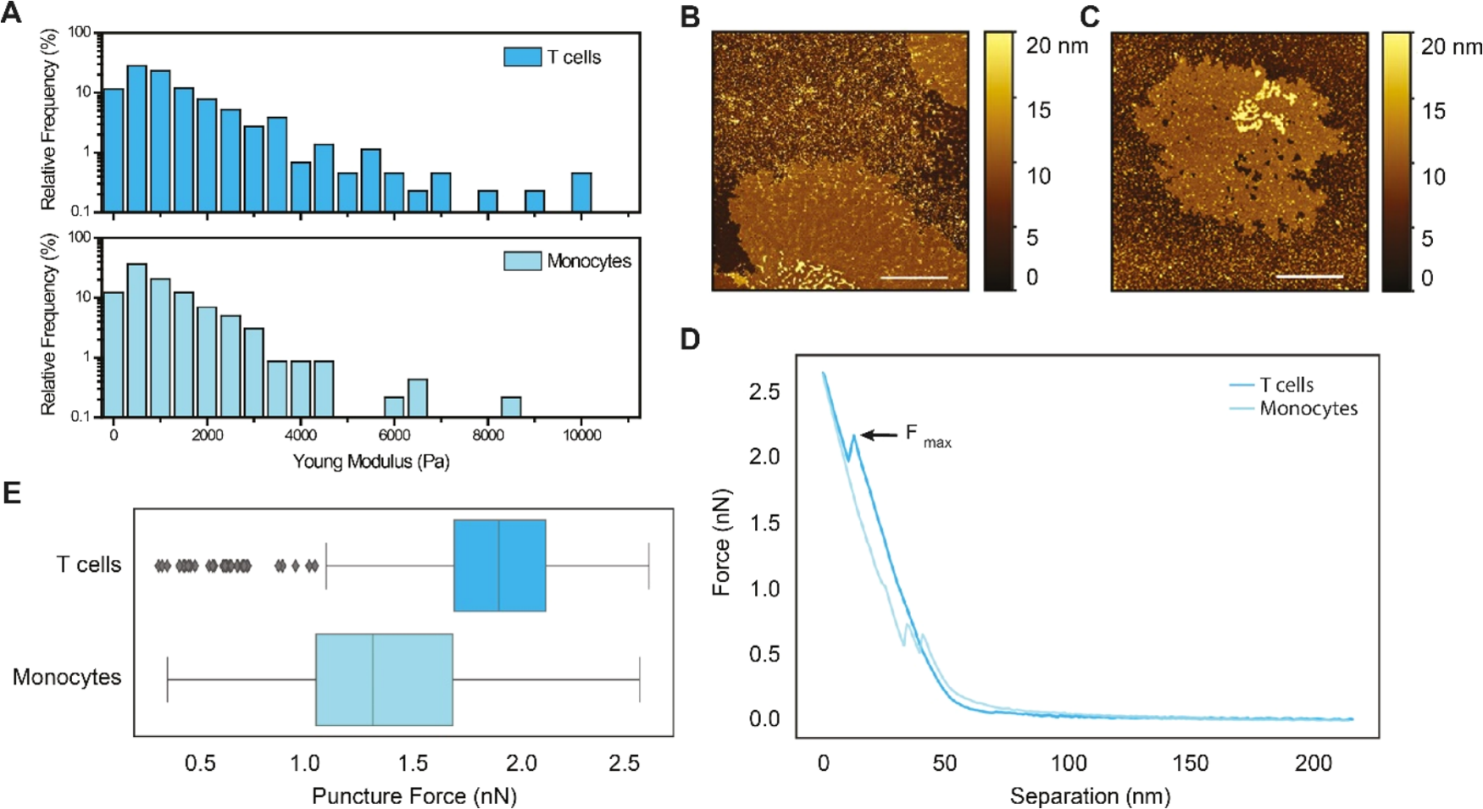
T cell membranes are more rigid than monocyte membranes. (A) Measurement of Young’s Modulus by AFM on intact GMPVs obtained from either T cells (dark blue) or monocytes (light blue). Data are presented as relative frequencies obtained from 4 replicates for each cell type and calculated with a bin width of 500 Pa. Statistical significance was calculated using one-way ANOVA; *p < 0.05. (B, C) Representative images of supported lipid bilayers from (B) T cells and (C) monocytes. Scale bars: 2 μm. (D) Representative force–separation curves measured on the supported lipid bilayer of T cell- and monocyte-derived GPMV populations. (E) Puncture force box plots for the T cell- and monocyte-derived GPMV populations. The box represents the first quartile (Q1) and the third quartile (Q3) of the data, with a line at the median. The whiskers extend from the box to the farthest data point lying within 1.5 × the interquartile range (IQR) from the box (Q1–1.5 × IQR, and Q3 + 1.5 × IQR). Outliers are represented by diamonds. The data presented was measured at an approach speed of 1 μm/s (*n* = 316 events for T cells and *n* = 308 events for monocytes). All data sets measured at different speeds and 2 different biological repetitions showed a similar significant trend.

We first used an AFM indentation assay on intact GPMVs obtained from both cell types to assess the mechanical deformability of the two membranes. Interestingly, we observed that the Young’s modulus of monocyte-derived GPMVs had a distribution mostly confined in the 0–4000 Pa range (Figures 6A and S16A), whereas the T cell-derived GMPV distribution extends into the 5000–10 000 Pa range (Figure S16B). This indicated that the T cell-derived GMPVs include a population of less deformable vesicles. These differences are in agreement with previous measurements of membrane rigidity obtained from primary immune cells;^59^ however, the mechanical properties of GPMVs can be affected by factors such as vesicle size, internal pressure, or shape variation. Thus, to directly measure the deformability of the membrane, we investigated whether T cell and monocyte membranes differ in mechanical properties using a puncture assay on supported lipid bilayers formed from GMPVs obtained from both cell types (Figure 6B,C).

Supported lipid bilayers locally collapse when they are pressed by the AFM tip. Typical puncture events are indicated on the force indentation curve by a dip when the tip penetrates the lipid layer. The force measured at this point represents the maximum force the membrane can endure (*F*_max_), and it directly relates to the deformability of the membrane. Puncture events have been detected in many supported lipid bilayers with various lipid compositions, as well as from supported natural membranes, providing direct insight into the mechanical stabilities of a variety of membranes.^16,60−62^

To form supported lipid bilayers from GPMVs, we deposited the vesicles on a Mg^2+^-modified mica surface and ruptured them by applying force with a fine tip (<10 nm radius). We subsequently identified areas where supported lipid bilayers had spread on the surface (Figure 6B,C), determined that a single bilayer was deposited, with an expected height around 5 nm (Figure S17A,B), and acquired force separation curves (Figure 6D). To objectively verify the presence of significant differences between cell types, the data were analyzed by supervised machine learning with a support vector machine model and 3-fold cross-validation. Under all conditions measured, the characteristic yield force for a puncture event differed significantly, with higher forces required to puncture T cell-derived GPMVs than monocyte-derived GPMVs (Figure 6E). Validation of the machine-learning data analysis demonstrated that T cells and monocytes are well-separated with a 70% average accuracy on 3-fold cross-validation with a support vector machine model (Figure S17C). This is direct evidence of differences in membrane compactness arising from compositional differences between the two populations, with T cell-derived GPMVs having markedly less deformable (or stiffer) membranes than monocyte-derived GPMVs, in agreement with recent reports indicating that T cells are stiffer on average than are monocytes.^59^

### Cholesterol Regulates the Balance between Endocytic and Membranal EV Entry by Affecting Membrane Stiffness

**Figure 7:**
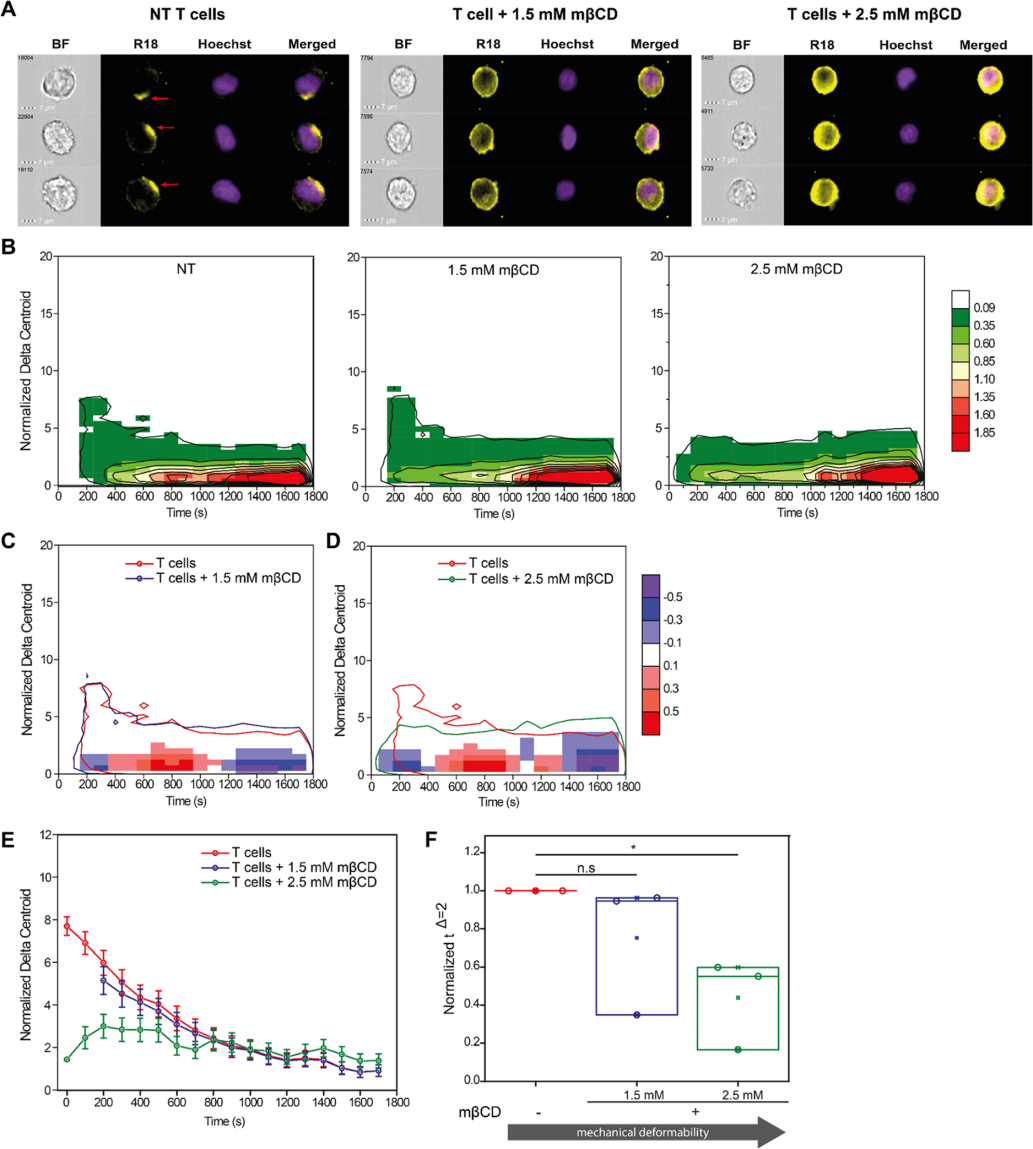
Removal of cholesterol shifts the uptake route of T cells from the membrane to the endocytic pathway. (A) Representative IFC images of untreated T cells, T cells treated with 1.5 mM mβCD, and T cells treated with 2.5 mM mβCD during the first 600 s after the addition of R18-labeled EVs. (B) Heat maps of average cellular R18 Δ_*xy*_^norm^ relative frequency after incubation of R18-labeled EVs with untreated T cells (left), cells treated with 1.5 mM mβCD (center), and cells treated with 2.5 mM mβCD (right). Heat map color represents the relative frequency (in percentage) at each specific Δ_*xy*_ - time coordinate, according to color legend in the figure. (C) Heat map of differences in Δ_*xy*_^norm^ events between untreated T cells and cells treated with 1.5 mM mβCD. (D) Heat map of differences in Δ_*xy*_^norm^ events between untreated T cells and cells treated with 2.5 mM mβCD. (E) Representative kinetic profile of Δ_*xy*_^norm^ for untreated T cells (red), T cells treated with 1.5 mM mβCD (blue), and T cells treated with 2.5 mM mβCD (green), showing a progressive loss of the polarized region at early time points. (F) Comparison of time to reach homogeneous fluorescence distribution (time to cross Δ_*xy*_^norm^ = 2) for untreated T cells (red), T cells treated with 1.5 mM mβCD (blue), and 2.5 mM mβCD (green), normalized to untreated T cells. All heat maps and bar graphs presented are based on 3 biological repeats. For heat maps of differences (panels C and D), blue areas indicate more events in the mβCD-treated cells compared to untreated T cells, and red areas indicate the opposite. Outlines indicate the isofrequency region at a relative frequency of 0.1% for either nontreated T cells (red), T cells treated with 1.5 mM mβCD (blue), and 2.5 mM mβCD (green). Each kinetic profile is presented as a weighted average (dot) and weighted standard error (whiskers). Heat map color in panels C and D represents the difference in relative frequency (in percentage) between untreated T cells and mβCD-treated T cells at each specific Δ_*xy*_^norm^ - time coordinates, according to color legend in the figure.

Since membrane tension and mechanical stiffness negatively regulate endocytosis,^63,64^ we sought to shift the membranal/endocytic balance of the recipient cells by modulating the membrane deformability. To that end, we treated T cells with mβCD, a compound that removes cholesterol from the plasma membrane,^65−68^ making it less rigid.^65^ T cells were treated with either 1.5 or 2.5 mM mβCD for 15 min as previously described.^69^ Using the Sytox viability assay,^70^ we confirmed that mβCD treatment did not alter cell viability (Figure S18). Next, the mβCD-treated T cells were incubated with R18-labeled *P. falciparum*-derived EVs, and the EV uptake was measured using IFC (Figures 7A and S19). Remarkably, upon cholesterol depletion, we did not observe the capping behavior at early time points, which we consistently observed for T cells not treated with mβCD; instead, for mβCD-treated T cells, there was a significant shift toward lower Δ*_xy_* frequencies (Figure 7A,B), which is characteristic of the monocyte uptake signal (Figure 2E). Comparison of the distributions of untreated T cells to cholesterol-depleted ones confirmed the qualitative observations: T cells treated with either 1.5 or 2.5 mM mβCD had increasingly fewer capping events than untreated T cells (Figure 7C,D). Treatment with 2.5 mM mβCD resulted in a clear reduction of Δ_*xy*_^norm^ in the early time points with a significant difference between untreated and cholesterol-depleted T cells (Figure 7D). The differences in the signal distribution between untreated T cells and 1.5 mM mβCD-treated T cells were negligible across the replicates; nevertheless, we still observe some individual reduction in the capped region, and at 2.5 mM mβCD, the Δ_*xy*_^norm^ distribution completely shifts from capped behavior at early times to a full uniform distribution (Figure S19). These data suggest that cholesterol depletion, which induces changes in the cell’s mechanical deformability, alters the mechanism of EV uptake.

Furthermore, analysis of the kinetic profiles revealed that EV uptake by untreated T cells starts in the polarized Δ_*xy*_^norm^ region (Figures 7E and S20) and progresses into the uniform region (Δ_*xy*_^norm^ < 2). In T cells treated with 1.5 mM mβCD, the kinetic profile was similar to that of untreated cells, whereas in cells treated with 2.5 mM mβCD, there was minimal fluorescence polarization (Figures 7E and S20). Estimation of time to reach homogeneous distribution (τ^Δ=2^) revealed that T cells more rapidly reached uniform fluorescence distribution with increasing concentration of mβCD (Figure 7F); there was a reduction in τ^Δ=2^ of 25 ± 35% for cells treated with 1.5 mM mβCD compared to untreated cells (weighted average ± weighted standard error, *n* = 3, *p* = 0.29) and of 56 ± 24% for cells treated with 2.5 mM mβCD compared to untreated cells (weighted average ± weighted standard error, *n* = 3, *p* = 0.015). Together, these data indicate that cholesterol in the plasma membrane regulates the mechanism of EV entry via modulation of the plasma membrane’s physical properties. Stiffer membranes are associated with direct membrane entry, and softer membranes favor endocytic entry. Removal of cholesterol, which reduces the rigidity of the plasma membrane, shifts the balance from the membranal toward endocytic entry of parasite-derived EVs.

**Figure 8:**
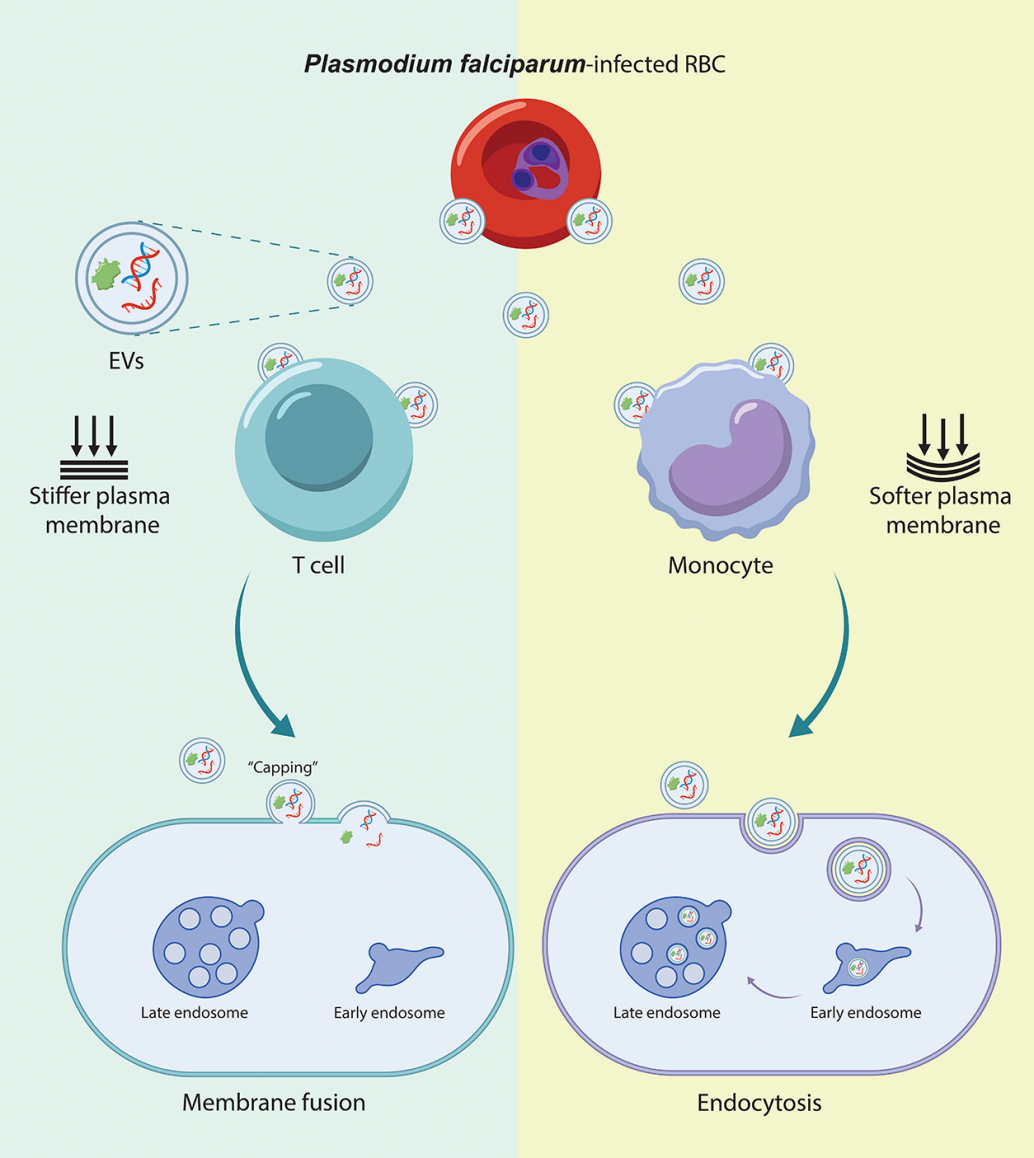
Proposed model for EV uptake into monocytes and T cells. EVs primarily enter monocytes through the endocytic pathway, whereas in T cells, uptake is mediated through specific regions of direct membrane contact or fusion.

Overall, this study suggests that despite the many differences in biomolecular profiles and metabolic activity between the immune cell types, the fundamental physical properties of membrane stiffness are sufficient to dictate where malaria-derived EVs preferential entry route would be, with a stiffer membrane favoring fusion at the plasma membrane and softer cells internalizing EVs via endocytosis (Figure 8).

## Conclusions

Previous research has suggested that EVs can enter cells through multiple mechanisms, including endocytosis and direct membrane fusion.^26,71^ The primary mode of entry for mammalian EVs is endocytic,^22,24,27,32^ but the uptake of malaria-derived EVs has not been well characterized. An interesting feature of parasite EVs is the wide range of recipient host cells to which the EVs are directed, including immune,^13^ endothelial cells,^17^ and naïve RBCs.^18^ RBCs lack the internal machinery responsible for EV endocytosis. Other factors have been shown to influence the EV uptake process, including membrane lipid and protein composition,^16,26^ and the mechanical properties of the EV membrane and the recipient host cell membrane.^72^ Thus, the mechanisms of uptake of EVs derived from *P. falciparum*-infected RBCs most likely depend on the type of target cells. Additionally, it was recently demonstrated that different subpopulations of *P. falciparum*-derived EVs are capable of spontaneously fusing with the plasma membrane and the early endosome.^16^ Here, by meticulously studying the biophysical mechanism underlying *P. falciparum*-derived EV uptake into two different human immune host cells, monocytes and T cells, we demonstrate that membrane mechanical properties play a significant role in the EV uptake mechanism.

Using assays that followed internalization dynamics of *P. falciparum*-derived EVs, labeled with R18, a self-quenching dye, into live cells, we compared uptake into cells of the innate and adaptive immune system (i.e., monocytes and T cells, respectively). When lipids labeled with R18 are incorporated into another membrane, the dye is diluted, which increases its fluorescence intensity.^46,47^ We demonstrated that T cells show a polarized or capped distribution pattern of membrane fluorescence, consistent with a local increase in R18 intensity due to EV membrane fusion events at the cell surface. In contrast, monocytes, which are phagocytic cells, show a uniform distribution pattern of the R18 signal post-EV uptake with distinct bright R18 intracellular puncta, as observed by confocal microscopy. This pattern and our finding that there was a much lower concentration of EVs in solution with monocytes compared to T cells after the same time of incubation are consistent with the endocytosis of EVs into monocytes. The faster uptake process in monocytes is characterized by extensive lipid recycling,^73^ albeit at a slower rate than internalization, with endocytic pits typically occurring within 1–2 min^74^ and the time frame of full membrane recycling approximately 10 min.^73^ This results in uniform and initially lower-intensity labeling distribution within monocytes compared with the T cells.

*P. falciparum*-derived EV uptake occurred with similar kinetics and distribution into GPMVs derived from both cell types. Therefore, the removal of the intracellular organelles from the recipient cells eliminated the uptake differences between the two cell types. Both results with live cells and GPMVs indicate that the capped pattern is due to interaction at the cell surface, namely, membrane fusion. Although our data does not reveal the structure or nature of the capped feature, both the initial fluorescence intensity at the plasma membrane, which is higher in T cells than monocytes, and the presence of polarized region on the membrane indicate that EV-cell interaction is at the surface and involves membrane fusion, as spontaneous transfer of free R18 molecules occur at a much slower rate than observed when R18 labels EVs.^75^ One additional aspect to consider is that the study of R18 dequenching cannot discriminate between full fusion (with successful cargo release within the cells) and hemifusion; the results we obtained, therefore, suggest that the preferential interaction of EVs with T cells occurs at the plasma membrane, but we cannot quantify the amount of cargo internalization. Our spectral flow cytometry and IFC analyses do indicate that cargo internalization for T cells occurs at a slower rate than internalization into monocytes, which supports our hypothesis that EV-mediated cargo delivery into T cells occurs through a more stochastic and less efficient membrane fusion process than the endocytosis-mediated delivery into monocytes.

We also demonstrated that modulation of the mechanical properties of T cell membranes by depletion of cholesterol resulted in a less rigid membrane compared to nontreated cells and led to a significant shift in the distribution pattern of R18 labeling. T cells with membranes lacking cholesterol had an EV uptake mechanism more similar to that of monocytes. Interestingly, the shift in the uptake route toward endocytosis occurred despite the possible inhibition of lipid-raft-mediated endocytosis caused by cholesterol depletion.^76^ This suggests that other endocytosis paths might be upregulated to compensate for the loss of lipid rafts, as has been previously reported.^77^ As we did not quantify the overall number of endocytic events but only focused on the route of EV internalization, further studies will be required to evaluate and identify the specific endocytic pathways involved in EV uptake.

EV uptake is dependent on the proteins and glycoproteins found on the surface of both the vesicle and the target cell.^24,26^ For example, proteinase K treatment of EVs derived from ovarian cancer cells significantly reduced their uptake into ovarian cancer cells,^22^ strongly supporting the role of proteins during the uptake process. It was also shown that mammalian EVs fuse with liposomes, mimicking the membrane composition of the late endosome in a pH- and protein-dependent manner.^78^ Chemical inhibitors and antibodies that block specific uptake pathways have been shown to inhibit EV entry into cells.^26^ Interestingly, it has been reported that the treatment of epithelial cells with mβCD results in significant inhibition of *Trichomonas vaginalis* EV uptake, specifically via lipid raft endocytosis,^72^ in contrast to what was observed in this study. In the case of *P. falciparum*-derived EVs, uptake by host monocytes also depends on N-glycoproteins, thus showing that terminal sialic acid on the N-glycans is essential for uptake by human monocytes.^79^

It should be noted that there is heterogeneity in both the EV populations and the target cell types in our study. Our focus here was on the biophysical properties of the recipient cell’s plasma membrane, but it will be of great interest in the future to understand the role of the biophysical properties of the EVs in dictating the uptake routes. It is possible that a population of EVs can simultaneously trigger several different uptake pathways, depending on the EV constituents and the properties of the host cells.^80−82^ A recent study demonstrated that two malaria-derived EV subsets with distinct membrane rigidities had distinct fusion specificities, supporting such a scenario.^16^ Overall, our results shed light on the importance of cell membrane mechanical properties in maintaining the balance among the different EV uptake mechanisms. Further research is needed before we have a comprehensive understanding of the roles of endocytic compartments and membrane affinity in the uptake process. For instance, quantifying EV binding to cell membranes and the subsequent fusion event could be achieved by combining secondary membrane labeling on the recipient cells and monitoring the evolution of the polarized signal. This would allow the determination of potential specific molecular partners on the cell surface, which have been found to directly correlate, for example, with the induction of membrane deformation and endocytosis of virions.^83^

In summary, advancing our understanding of *P. falciparum-*derived EV uptake mechanisms provides deeper insights into disease progression and the virulence strategies developed by the deadliest malaria parasites. Such knowledge will aid in the design of novel and sophisticated drug delivery systems using engineered EVs. There is considerable interest in utilizing EVs to enhance vaccine delivery. It was recently shown that *Plasmodium yoelii* EVs can be used to immunize mice against lethal infection.^84^ Understanding how to modulate such EVs, either engineered or collected from parasites, to reach specific immune cell targets will provide invaluable information that will guide the development of better prevention strategies and improve immunization outcomes in the most afflicted regions of the world.

## Methods

### *P. falciparum* Culture

*P. falciparum* parasites (NF54 strain was kindly provided by the Malaria Research Reference Reagent Resource Center) were cultured in human RBCs using a standard method.^85^ Briefly, parasites were grown at 4% hematocrit in pooled healthy uninfected RBCs, provided by the Magen David Adom and Sheba Medical Center Blood Bank Laboratories, Israel (IRB—1634-1), at 37 °C in a gas mixture of 1% O_2_ and 5% CO_2_ in N_2_ in RPMI 1640 medium, pH 7.4 (Diagnovum, Cat#: D840-P10), supplemented with 25 mg/mL 4-(2-hydroxyethyl)-1-piperazineethanesulfonic acid (Sigma-Aldrich, Cat#: H3375), 50 μg/mL hypoxanthine (Sigma-Aldrich, Cat#: 4010CBC), 2 mg/mL sodium bicarbonate (Fischer Scientific, Cat#: 15588134), 20 μg/mL gentamycin (Sigma-Aldrich, Cat#: G9654), and 0.5% AlbumaxII (Gibco, Cat#: 11021045). Parasite growth was monitored by Giemsa staining (Sigma-Aldrich, catalog no. 109204) of methanol-fixed blood smears. Cultures were tested for mycoplasma infections twice a month using a commercial kit (MycoAlert Plus Lonza, Cat#: LT07–318).

### Human THP-1 Monocyte Culture

THP-1 cells were cultured as previously described.^86^ Briefly, cells were grown in complete RPMI 1640+ with l-glutamine (Biological Industries Ltd., Cat#: 011001A) supplemented with 10% FBS (Sigma-Aldrich, Cat#: F7524) and PenStrep (Diagnovum, Cat#: D910) in a humidified incubator at 37 °C with 5% CO_2_. Cells were tested for mycoplasma once a month using a commercial kit MycoAlert Plus (Lonza, Cat#: LT07–318).

### Human Jurkat Cell Culture

The Jurkat E6–1 T cells were cultured as previously described.^87,88^ In brief, cells were grown in complete RPMI 1640+ with l-glutamine, 10% FBS, and PenStrep in a humidified incubator at 37 °C and 5% CO_2_. Cells were tested for mycoplasma once a month.

### EV Isolation

Growth media was collected from high parasitemia (≥5%) *P. falciparum-*infected RBC culture. Prior to media collection, cultures were tightly synchronized using 5% sorbitol (Sigma-Aldrich, Cat#: S1876) according to a standard protocol.^5^ EV purification was performed as previously described.^18^ Briefly, the medium was spun down at 413*g* for 5 min. The remaining cells were cleared by an additional centrifugation at 413*g* for 5 min, followed by a centrifugation at 1900*g* for 10 min. To eliminate cell debris, the media was then centrifuged at 15 180*g* for 1 h at 4 °C. The supernatant was filtered through a 450 nm pore filter, and EVs were concentrated using a Vivacell 100 with a 100 kDa cutoff (Sartorius AG, Cat#: VC1042) as described in the manufacturer’s protocol. Pelleted EVs were obtained as previously described^89^ via 150 000*g* ultracentrifugation for 16 h at 4 °C using a Beckman OPTIMA90X ultracentrifuge with a Ti70 rotor (Beckman Coulter). Finally, the pellet containing EVs was carefully suspended in PBS containing Ca^2+^ and Mg^2+^ (Biological Industries) for further analysis.

### EV Labeling and Purification

EVs were incubated with thiazole orange (TO, Sigma-Aldrich, Cat#: 390062), which binds to RNA^38^ at a 1:500 dilution at 37 °C for 30 min. Unlabeled EVs were used as a control. EVs were pelleted by overnight ultracentrifugation at 4 °C and 150 000*g* (Beckman OPTIMA90X ultracentrifuge with a Ti70 rotor). The pellet was then carefully suspended in PBS containing Ca^2+^ and Mg^2+^ for further analysis.

For octadecyl rhodamine B (R18) labeling of EV membranes, resuspended EVs were incubated with R18 (Sigma-Aldrich, cat. no. 83685) at a dilution of 1:100 at 37 °C for 30 min. Unlabeled EVs were used as a control. EVs were slowly loaded over a 2.5 mL sterile 20% sucrose solution (prepared in PBS containing Ca^2+^ and Mg^2+^), forming a layer. EVs were pelleted by ultracentrifugation using a swinging bucket rotor (Beckman Coulter) at 102 400*g* for 4 h at 4 °C. The supernatant was discarded, and the EVs were resuspended in sterile PBS containing Ca^2+^ and Mg^2+^ for further analysis.

### Poly-d-lysine-Coated Plates

Plates were coated with poly-d-lysine (Gibco, Cat#: A3890401) as per the manufacturer’s protocol. Briefly, 300 μL of poly-d-lysine solution was added to each well of 24-well-plates and left overnight at room temperature. Next, plates were washed three times with doubly distilled water and dried completely. Monocytes or T cells were plated in the coated plates at 1 × 10^6^ cells/well and incubated at 37 °C with 5% CO_2_ overnight to allow complete adherence of the cells to the plate.

### GPMV Isolation

GPMVs were prepared as previously described.^90^ In brief, monocytes and T cells plated on poly-d-lysine-coated 24-well-plates were washed with a GPMV vesiculation buffer containing 20 mM HEPES, 150 mM NaCl, and 2 mM CaCl_2_ in PBS (Ca^2+^-/Mg^2+^-) at pH 7.4 to remove any detached cells. For labeling of GPMV membranes, 2 μg/mL of lipophilic tracer Dil (Thermo Fisher, Cat#: D282) was added to the buffer, and after incubation at 37 °C with 5% CO_2_ for 30 min, cells were washed three times with GPMV vesiculation buffer to remove excess dye. To induce vesiculation, monocytes and T cells were incubated for at least 2 h at 37 °C with 5% CO_2_ in 200 μL of active vesiculation buffer containing 2 mM dithiothreitol (DTT, Cat#: DB0058) and 27.6 mM formaldehyde (J.T. Baker, Cat#: UN2209). The GPMVs were collected and centrifuged at 200 g for 5 min to remove the remaining cells and cell debris. GPMVs were kept at 4 °C. DiI-labeled GPMVs were employed only for the purpose of visualization of the GPMVs. For all experiments involving the incubation of GPMVs with R18-labeled EVs, unlabeled GPMVs were utilized.

### Nanoparticle Tracking Analysis

Vesicle size distributions and concentrations were calculated using nanoparticle tracking analysis^91^ with the NanoSight NS300 device (Malvern Panalytical Ltd.) using a 405 nm filter. Sample size distributions were calibrated in a liquid suspension by the analysis of Brownian motion via light scattering, and the sizes of the particles were estimated based on their hydrodynamic radii.^91^

### Kinetic Analysis of EV Uptake into Live Cells Using Multispectral Imaging Flow Cytometry Analysis

For kinetic analyses of uptake of EVs, 1 × 10^6^ monocytes or T cells were labeled with 4 μM Hoechst (Life Technologies, cat. no. 62249) at a dilution of 1:8000. The cells were incubated with the dye for 10 min, followed by two washes with PBS containing Ca^2+^ and Mg^2+^. The Hoechst-labeled cells were kept on ice until assayed. For DTT/formaldehyde treatment, cells were incubated for 20 min at 37 °C with 5% CO_2_ in 2 mL of active vesiculation buffer with 2 mM DTT and 27.6 mM formaldehyde. Cells were then washed with PBS containing Ca^2+^ and Mg^2+^ and placed on ice. R18-labeled or TO-labeled *P. falciparum*-derived EVs were added to the cells, and the EV uptake was assessed using IFC for 30 min. Cells were imaged using a multispectral ImageStreamX Mark II imaging flow cytometer (Amnis Corp.) using a 60× lens (NA = 0.9). The lasers used were 405 nm (50 mW) and 561 nm (120 mW), and the channels collected were bright field (Ch01 and Ch09), TO (Ch02), R18 (Ch03), and Hoechst (Ch07). At least 20 000 cells were collected from each sample. Data was analyzed using the manufacturer’s image analysis software (IDEAS 6.3; Amnis Corp.). Monocytes and T cells were gated for (a) single cells using the area and aspect ratio features and (b) focused cells using the Gradient RMS feature as previously described.^92^ Cropped cells were eliminated by plotting the cell area of the bright-field image against the Centroid X feature (the number of pixels in the horizontal axis from the left corner of the image to the center of the cell mask). Vesicle internalization was evaluated by considering the intensity (the sum of the background-subtracted pixel values within the masked area of the image) and the max pixel (the largest value of the background-subtracted pixel). The delta centroid (Δ*_xy_*) value was measured as the distance from the center of the cell (center of the bright-field mask) to the intensity-weighted center of the membranal labeling (Ch03) normalized to a 0 to 1 scale (0 being the geometrical center of the cell and 1 being the edges of the mask). In cases where labeling was uniformly distributed throughout the cellular membrane, the radial Δ*_xy_* values were small. When the labeling was localized to a certain area of the membrane, the radial Δ_*xy*_ values were large. For GPMVs, a similar analysis was performed following their isolation from the cells.

### Spectral Flow Cytometry

To evaluate the uptake of fluorescently labeled *P. falciparum*-derived EVs, monocytes or T cells were plated into wells of a 24-well-plate on the day of the experiment at 1 × 10^6^ cells/well for analysis of TO-labeled EV uptake or 0.25 × 10^6^ for the R18-labeled EV uptake. The lower number of cells was due to the additional purification step required for the R18-labeled EVs. TO-labeled EVs were introduced into the cells at a ratio of 10 000 or 30 000 EVs/cell, and R18-labeled EVs were added to the cells at 10 000 EVs/cell, and the cells were incubated at 37 °C with 5% CO_2_ for 5, 15, or 30 min. Next, cells were centrifuged at 1300 rpm for 5 min, and the supernatant was discarded. The cell pellets were gently resuspended in ice-cold flow cytometry buffer (PBS containing Ca^2+^ and Mg^2+^, 1 mM EDTA, and 2% FBS). Uptake was analyzed by spectral flow cytometry using the Aurora Spectral Flow Cytometer (Cytek Biosciences). The instrument is equipped with 5 lasers and 64 detection channels. During acquisition sessions, 20 000 cells from each sample were collected for further analysis and comparisons. The data acquisition and deconvolution, including autofluorescence detection and subtraction, was performed using the SpectroFlo Software v3.3.0. Plots of time- and EV concentration-dependent changes in cellular uptake were created using FlowJo software (v10.8.1). The *XY* plots and statistical analyses were carried out using GraphPad Prism v10.2.0 software.

### AFM

GPMV suspensions were placed on Petri dishes coated with Concanavalin A-coated (Sigma-Aldrich, Cat#: C5275) overnight to allow adsorption. Prior to scanning, 2 mL of GPMV vesiculation buffer was added to the sample. Imaging was performed with a JPK Nanowizard III AFM microscope (Bruker Nano GmbH) in the QI mode. In this mode, force–distance curves are recorded at each pixel and are used to acquire topographic images and nanomechanical data simultaneously. Measurements were conducted with a qp-BioAC-CI CB3 probe (Nanosensors), spring constant ≈0.06 N/m. The spring constant was measured prior to each measurement by using the contact-free spring calibration method in the JPK software v.6. Force curves from the center of each GPMV were used to calculate the elastic modulus by applying a contact mechanical Hertzian model (using JPK data processing software version 6.1.86), with a Poisson ratio of 0.5 and a conical tip with an opening half angle of 22 degrees. Only vesicles with heights between 2 and 6 μm were included in the analysis. For the mechanical measurements, images of 60 × 60 μm^2^ were captured at 80 × 80 pixel resolution. The force applied to each pixel was 140 pN, and the approach speed was 30 μm/s. Image analysis was performed using Gwyddion^93^ and JPK-SPM data processing software, version 6.2.172.

### Supported Membrane Indentation Assay

GPMVs suspended in PBS containing Ca^2+^ and Mg^2+^ were placed on Mg-modified mica (freshly cleaved mica incubated with MgCl_2_ solution for 2 min and then washed with vesiculation buffer). Vesicles were imaged with a qp-BioAC equipped with a CB3 probe with a tip radius of curvature smaller than 10 nm (Nanosensors) in QI mode while applying 1 nN force to cause vesicle puncture. A zoomed area was imaged in order to find an area with a supported lipid bilayer spread on the surface. Hundreds of force–distance curves were recorded in different locations in each bilayer patch, and each made over a 7 × 7 grid in a 1 × 1 μm^2^ area. The same analysis was performed on a control area without a bilayer.

The obtained force curves were subjected to puncture event analysis using functions from the scientific computing library Scipy^94^ (version 1.9.1). Puncture forces were obtained by finding the local minima and maxima of each curve. A puncture was detected if the maximum–minimum difference was above 25 pN and if the minimum was found in the force curve region with a force above 250 pN.

To evaluate the separation between T cell and monocyte populations by their puncture forces into an accuracy metric, a machine learning model was applied with 3-fold cross-validation. To address imbalance in the data, the majority group was down-sampled to the same number of instances as in the minority group. A support vector machine model was chosen with a radial basis function kernel. An average accuracy of ∼70% was obtained. The scikit-learn library for machine learning in Python was used.^95^

### Cholesterol Depletion Assay

Cholesterol depletion was performed as previously described.^69^ Briefly, 1 × 10^6^ T cells were treated with 1.5 or 2.5 mM methyl-β-cyclodextrin (mβCD; Sigma-Aldrich, Cat#: M7439) and incubated at 37 °C with 5% CO_2_ for 15 min. Untreated cells were used as a control. Next, the cells were washed with PBS containing Ca^2+^ and Mg^2+^. Cells were kept on ice prior to the addition of R18-labeled EVs. Samples were imaged immediately after EV addition in a multispectral IFC for 30 min, as described above.

### Confocal Imaging

Samples were imaged using a spinning disk confocal (Yokogawa CSU-W1) microscope with a 50 μm pinhole (Nikon). Images were acquired with a CFI Plan Apochromat 60× oil objective (N.A-1.45) and DAPI and RFP filters and with an sCMOS camera (Photometrics, PRIME – BSI). Images were collected in Z-stack mode with 0.5 μm steps through 15–20 μm. Images were analyzed by using ImageJ software.

### Analysis of R18 Fluorescence Using Imaging Flow Cytometry

R18 total intensity and Δ*_xy_* between the R18 channel (membranal fluorescence) and bright-field channel (cell center of mass) from IFC were time-truncated to the lowest common time point across all repeats and conditions (in this case, 1750 s was selected). Subsequently, each data set was normalized to its respective baseline median calculated over the last 100 s of acquisition. Both steps were performed by using a MATLAB script. The resulting time-truncated and median-normalized data sets were then 2D-binned both in either Δ_*xy*_^norm^ (from 0 to 20 bin centers, bin width 0.5) or total normalized R18 intensity (from 0 to 1 bin centers, bin width 0.1) and time (from 0 to 1800 s bin centers, bin width 100 s), and the relative frequency was calculated for each 2D-bin cell using OriginPro software.

### Kinetic Profiles of R18 Normalized Fluorescence Intensity and Δ_*xy*_^norm^

Kinetic profiles for each data set were obtained by first filtering the obtained 2D-bin frequency matrices for any value below random noise (estimated as the frequency corresponding to homogeneous distribution −0.12 Δ_*xy*_^norm^ and 0.23 for normalized intensity), thus obtaining a filtered frequency matrix. Subsequently, a 2D-bin matrix with the median value for each cell at the same binning interval was calculated for each data set. For each time bin center, the weighted average of the medians of either Δ_*xy*_^norm^ or total normalized R18 intensity was calculated by using the filtered relative frequency matrix as weights. The standard error for each time bin center was calculated by using the weighted standard deviation with relative weights for each time bin.

### Statistical Analysis

For all comparisons, either a two-sample *t*-test or one-way ANOVA test was performed between relative frequencies with statistical significance *p* < 0.05 using a MATLAB script.

## Supporting Information Available

Characterization (NTA, cryoEM, AFM) of *P. falciparum*-derived EVs (Figure S1); technical repeats and control experiments of R18-labeled EVs uptake and data analysis for all conditions presented in the main text (Figures S2–S6, S12–S14, S19, S20); analysis of R18 and TO-labeled EVs uptake kinetics with spectral flow cytometry (Figures S8 and S9); AFM and imaging flow cytometry characterization of GPMVs (Figures S10 and S11); uptake kinetics and data analysis for cells treated with DTT (Figure S15); AFM measurement of cell membrane Young modulus (Figure S16) and indentation profiles (Figure S17); and cell viability following incubation with mβCD (Figure S18) (PDF)

## References

[ref1] World Malaria Report 2023. https://www.who.int/teams/global-malaria-programme/reports/world-malaria-report-2023. (accessed April 18, 2024).

[ref2] Ofir-Birin Y.; Heidenreich M.; Regev-Rudzki N. Pathogen-Derived Extracellular Vesicles Coordinate Social Behaviour and Host Manipulation. Semin. Cell Dev. Biol. 2017, 67, 83–90. 10.1016/j.semcdb.2017.03.004. 28366828

[ref3] Mashburn L. M.; Whiteley M. Membrane Vesicles Traffic Signals and Facilitate Group Activities in a Prokaryote. Nature 2005, 437 (7057), 422–425. 10.1038/nature03925. 16163359

[ref4] van der Pol E.; Böing A. N.; Harrison P.; Sturk A.; Nieuwland R. Classification, Functions, and Clinical Relevance of Extracellular Vesicles. Pharmacol. Rev. 2012, 64 (3), 676–705. 10.1124/pr.112.005983. 22722893

[ref5] Sisquella X.; Ofir-Birin Y.; Pimentel M. A.; Cheng L.; Abou Karam P.; Sampaio N. G.; Penington J. S.; Connolly D.; Giladi T.; Scicluna B. J.; Sharples R. A.; Waltmann A.; Avni D.; Schwartz E.; Schofield L.; Porat Z.; Hansen D. S.; Papenfuss A. T.; Eriksson E. M.; Gerlic M.; Hill A. F.; Bowie A. G.; Regev-Rudzki N. Malaria Parasite DNA-Harbouring Vesicles Activate Cytosolic Immune Sensors. Nat. Commun. 2017, 8 (1), 1985 10.1038/s41467-017-02083-1. 29215015 PMC5719353

[ref6] De Gassart A.; Géminard C.; Février B.; Raposo G.; Vidal M. Lipid Raft-Associated Protein Sorting in Exosomes. Blood 2003, 102 (13), 4336–4344. 10.1182/blood-2003-03-0871. 12881314

[ref7] Thorsteinsson K.; Olsén E.; Schmidt E.; Pace H.; Bally M. FRET-Based Assay for the Quantification of Extracellular Vesicles and Other Vesicles of Complex Composition. Anal. Chem. 2020, 92 (23), 15336–15343. 10.1021/acs.analchem.0c02271. 33179908 PMC7735656

[ref8] Buzás E. I.; Tóth E.; Sódar B. W.; Szabó-Taylor K. Molecular Interactions at the Surface of Extracellular Vesicles. Semin. Immunopathol. 2018, 40 (5), 453–464. 10.1007/s00281-018-0682-0. 29663027 PMC6208672

[ref9] Dong G.; Filho A. L.; Olivier M. Modulation of Host-Pathogen Communication by Extracellular Vesicles (EVs) of the Protozoan Parasite Leishmania. Front. Cell. Infect. Microbiol. 2019, 9, 100 10.3389/fcimb.2019.00100. 31032233 PMC6470181

[ref10] Bonsergent E.; Grisard E.; Buchrieser J.; Schwartz O.; Théry C.; Lavieu G. Quantitative Characterization of Extracellular Vesicle Uptake and Content Delivery within Mammalian Cells. Nat. Commun. 2021, 12 (1), 1864 10.1038/s41467-021-22126-y. 33767144 PMC7994380

[ref11] Mantel P. Y.; Marti M. The Role of Extracellular Vesicles in Plasmodium and Other Protozoan Parasites. Cell Microbiol. 2014, 16 (3), 344–354. 10.1111/cmi.12259. 24406102 PMC3965572

[ref12] Regev-Rudzki N.; Wilson D. W.; Carvalho T. G.; Sisquella X.; Coleman B. M.; Rug M.; Bursac D.; Angrisano F.; Gee M.; Hill A. F.; Baum J.; Cowman A. F. Cell-Cell Communication between Malaria-Infected Red Blood Cells via Exosome-like Vesicles. Cell 2013, 153 (5), 1120–1133. 10.1016/j.cell.2013.04.029. 23683579

[ref13] Mantel P. Y.; Hoang A. N.; Goldowitz I.; Potashnikova D.; Hamza B.; Vorobjev I.; Ghiran I.; Toner M.; Irimia D.; Ivanov A. R.; Barteneva N.; Marti M. Malaria-Infected Erythrocyte-Derived Microvesicles Mediate Cellular Communication within the Parasite Population and with the Host Immune System. Cell Host Microbe 2013, 13 (5), 521–534. 10.1016/j.chom.2013.04.009. 23684304 PMC3687518

[ref14] Ofir-Birin Y.; Ben Ami Pilo H.; Cruz Camacho A.; Rudik A.; Rivkin A.; Revach O. Y.; Nir N.; Block Tamin T.; Abou Karam P.; Kiper E.; Peleg Y.; Nevo R.; Solomon A.; Havkin-Solomon T.; Rojas A.; Rotkopf R.; Porat Z.; Avni D.; Schwartz E.; Zillinger T.; Hartmann G.; Di Pizio A.; Quashie N.; Ben; Dikstein R.; Gerlic M.; Torrecilhas A. C.; Levy C.; Nolte-‘t Hoen E. N. M.; Bowie A. G.; Regev-Rudzki N. Malaria Parasites Both Repress Host CXCL10 and Use It as a Cue for Growth Acceleration. Nat. Commun. 2021, 12 (1), 4851 10.1038/s41467-021-24997-7. 34381047 PMC8357946

[ref15] Ye W.; Chew M.; Hou J.; Lai F.; Leopold S. J.; Loo H. L.; Ghose A.; Dutta A. K.; Chen Q.; Ooi E. E.; White N. J.; Dondorp A. M.; Preiser P.; Chen J. Microvesicles from Malaria-Infected Red Blood Cells Activate Natural Killer Cells via MDA5 Pathway. PLoS Pathog. 2018, 14 (10), e1007298 10.1371/JOURNAL.PPAT.1007298. 30286211 PMC6171940

[ref16] Abou Karam P.; Rosenhek-Goldian I.; Ziv T.; Ben Ami Pilo H.; Azuri I.; Rivkin A.; Kiper E.; Rotkopf R.; Cohen S. R.; Torrecilhas A. C.; Avinoam O.; Rojas A.; Morandi M. I.; Regev-Rudzki N. Malaria Parasites Release Vesicle Subpopulations with Signatures of Different Destinations. EMBO Rep. 2022, 23 (7), e54755 10.15252/embr.202254755. 35642585 PMC9253735

[ref17] Mantel P. Y.; Hjelmqvist D.; Walch M.; Kharoubi-Hess S.; Nilsson S.; Ravel D.; Ribeiro M.; Grüring C.; Ma S.; Padmanabhan P.; Trachtenberg A.; Ankarklev J.; Brancucci N. M.; Huttenhower C.; Duraisingh M. T.; Ghiran I.; Kuo W. P.; Filgueira L.; Martinelli R.; Marti M. Infected Erythrocyte-Derived Extracellular Vesicles Alter Vascular Function via Regulatory Ago2-MiRNA Complexes in Malaria. Nat. Commun. 2016, 7 (1), 12727 10.1038/ncomms12727. 27721445 PMC5062468

[ref18] Dekel E.; Yaffe D.; Rosenhek-Goldian I.; Ben-Nissan G.; Ofir-Birin Y.; Morandi M. I.; Ziv T.; Sisquella X.; Pimentel M. A.; Nebl T.; Kapp E.; Ohana Daniel Y.; Karam P. A.; Alfandari D.; Rotkopf R.; Malihi S.; Temin T. B.; Mullick D.; Revach O. Y.; Rudik A.; Gov N. S.; Azuri I.; Porat Z.; Bergamaschi G.; Sorkin R.; Wuite G. J. L.; Avinoam O.; Carvalho T. G.; Cohen S. R.; Sharon M.; Regev-Rudzki N. 20S Proteasomes Secreted by the Malaria Parasite Promote Its Growth. Nat. Commun. 2021, 12 (1), 1172 10.1038/s41467-021-21344-8. 33608523 PMC7895969

[ref19] Abdi A.; Yu L.; Goulding D.; Rono M. K.; Bejon P.; Choudhary J.; Rayner J. Proteomic Analysis of Extracellular Vesicles from a Plasmodium Falciparum Kenyan Clinical Isolate Defines a Core Parasite Secretome. Wellcome Open Res. 2017, 2, 50 10.12688/wellcomeopenres.11910.1. 28944300 PMC5583745

[ref20] Kioko M.; Pance A.; Mwangi S.; Goulding D.; Kemp A.; Rono M.; Ochola-Oyier L. I.; Bull P. C.; Bejon P.; Rayner J. C.; Abdi A. I. Extracellular Vesicles Could Be a Putative Posttranscriptional Regulatory Mechanism That Shapes Intracellular RNA Levels in Plasmodium Falciparum. Nat. Commun. 2023, 14 (1), 6447 10.1038/s41467-023-42103-x. 37833314 PMC10575976

[ref21] Rivkin A.; Ben-Hur S.; Regev-Rudzki N. Malaria Parasites Distribute Subversive Messages across Enemy Lines. Trends Parasitol. 2017, 33 (1), 2–4. 10.1016/j.pt.2016.11.005. 27889370

[ref22] Escrevente C.; Keller S.; Altevogt P.; Costa J. Interaction and Uptake of Exosomes by Ovarian Cancer Cells. BMC Cancer 2011, 11 (1), 108 10.1186/1471-2407-11-108. 21439085 PMC3072949

[ref23] Gurung S.; Perocheau D.; Touramanidou L.; Baruteau J. The Exosome Journey: From Biogenesis to Uptake and Intracellular Signalling. Cell Commun. Signaling 2021, 19 (1), 47 10.1186/s12964-021-00730-1. PMC8063428 33892745

[ref24] Friese C.; Yang J. Extracellular Vesicle Transportation and Uptake by Recipient Cells Physiol. Behav. 2019; Vol. 46 (2), , pp 248–256 10.3390/pr9020273.Extracellular.

[ref25] Raposo G.; Stoorvogel W. Extracellular Vesicles: Exosomes, Microvesicles, and Friends. J. Cell Biol. 2013, 200, 373–383. 10.1083/jcb.201211138. 23420871 PMC3575529

[ref26] Mulcahy L. A.; Pink R. C.; Carter D. R. F. Routes and Mechanisms of Extracellular Vesicle Uptake. J. Extracell. Vesicles 2014, 3 (1), 24641 10.3402/jev.v3.24641. PMC4122821 25143819

[ref27] Horibe S.; Tanahashi T.; Kawauchi S.; Murakami Y.; Rikitake Y. Mechanism of Recipient Cell-Dependent Differences in Exosome Uptake. BMC Cancer 2018, 18 (1), 47 10.1186/s12885-017-3958-1. 29306323 PMC5756423

[ref28] Montecalvo A.; Larregina A. T.; Shufesky W. J.; Stolz D. B.; Sullivan M. L. G.; Karlsson J. M.; Baty C. J.; Gibson G. A.; Erdos G.; Wang Z.; Milosevic J.; Tkacheva O. A.; Divito S. J.; Jordan R.; Lyons-Weiler J.; Watkins S. C.; Morelli A. E. Mechanism of Transfer of Functional MicroRNAs between Mouse Dendritic Cells via Exosomes. Blood 2012, 119 (3), 756–766. 10.1182/blood-2011-02-338004. 22031862 PMC3265200

[ref29] Morelli A. E.; Larregina A. T.; Shufesky W. J.; Sullivan M. L. G.; Stolz D. B.; Papworth G. D.; Zahorchak A. F.; Logar A. J.; Wang Z.; Watkins S. C.; Falo L. D.; Thomson A. W. Endocytosis, Intracellular Sorting, and Processing of Exosomes by Dendritic Cells. Blood 2004, 104 (10), 3257–3266. 10.1182/blood-2004-03-0824. 15284116

[ref30] Joshi B. S.; de Beer M. A.; Giepmans B. N. G.; Zuhorn I. S. Endocytosis of Extracellular Vesicles and Release of Their Cargo from Endosomes. ACS Nano 2020, 14 (4), 4444–4455. 10.1021/acsnano.9b10033. 32282185 PMC7199215

[ref31] Eguchi S.; Takefuji M.; Sakaguchi T.; Ishihama S.; Mori Y.; Tsuda T.; Takikawa T.; Yoshida T.; Ohashi K.; Shimizu Y.; Hayashida R.; Kondo K.; Bando Y. K.; Ouchi N.; Murohara T. Cardiomyocytes Capture Stem Cell-Derived, Anti-Apoptotic MicroRNA-214 via Clathrin-Mediated Endocytosis in Acute Myocardial Infarction. J. Biol. Chem. 2019, 294 (31), 11665–11674. 10.1074/jbc.RA119.007537. 31217281 PMC6682734

[ref32] Zheng Y.; Tu C.; Zhang J.; Wang J. Inhibition of Multiple Myeloma-derived Exosomes Uptake Suppresses the Functional Response in Bone Marrow Stromal Cell. Int. J. Oncol. 2019, 54 (3), 1061–1070. 10.3892/ijo.2019.4685. 30664188

[ref33] Feng D.; Zhao W. L.; Ye Y. Y.; Bai X. C.; Liu R. Q.; Chang L. F.; Zhou Q.; Sui S. F. Cellular Internalization of Exosomes Occurs through Phagocytosis. Traffic 2010, 11 (5), 675–687. 10.1111/j.1600-0854.2010.01041.x. 20136776

[ref34] Fabbri M.; Paone A.; Calore F.; Galli R.; Gaudio E.; Santhanam R.; Lovat F.; Fadda P.; Mao C.; Nuovo G. J.; Zanesi N.; Crawford M.; Ozer G. H.; Wernicke D.; Alder H.; Caligiuri M. A.; Nana-Sinkam P.; Perrotti D.; Croce C. M. MicroRNAs Bind to Toll-like Receptors to Induce Prometastatic Inflammatory Response. Proc. Natl. Acad. Sci. U.S.A. 2012, 109 (31), E2110–E2116. 10.1073/pnas.1209414109. 22753494 PMC3412003

[ref35] Parolini I.; Federici C.; Raggi C.; Lugini L.; Palleschi S.; De Milito A.; Coscia C.; Iessi E.; Logozzi M.; Molinari A.; Colone M.; Tatti M.; Sargiacomo M.; Fais S. Microenvironmental PH Is a Key Factor for Exosome Traffic in Tumor Cells. J. Biol. Chem. 2009, 284 (49), 34211–34222. 10.1074/jbc.M109.041152. 19801663 PMC2797191

[ref36] Carrera-Bravo C.; Koh E. Y.; Tan K. S. W. The Roles of Parasite-Derived Extracellular Vesicles in Disease and Host-Parasite Communication. Parasitol. Int. 2021, 83, 102373 10.1016/j.parint.2021.102373. 33933651

[ref37] Dekel E.; Abou Karam P.; Ohana-daniel Y.; Biton M.; Regev-rudzki N. Antibody-free labeling of malaria-derived extracellular vesicles using flow cytometry. Biomedicines 2020, 8 (5), 98 10.3390/biomedicines8050098. 32349226 PMC7277110

[ref38] Alfandari D.; Ben Ami Pilo H.; Abou Karam P.; Dagan O.; Joubran C.; Rotkopf R.; Regev-Rudzki N.; Porat Z. Monitoring Distribution Dynamics of EV RNA Cargo Within Recipient Monocytes and Macrophages. Front. Cell. Infect. Microbiol. 2022, 11, 739628 10.3389/fcimb.2021.739628. 35155269 PMC8825493

[ref39] Janmey P. A.; Kinnunen P. K. J. Biophysical Properties of Lipids and Dynamic Membranes. Trends Cell Biol. 2006, 16 (10), 538–546. 10.1016/j.tcb.2006.08.009. 16962778

[ref40] Joseph J. G.; Liu A. P. Mechanical Regulation of Endocytosis: New Insights and Recent Advances. Adv. Biosyst. 2020, 4 (5), 1900278 10.1002/adbi.201900278. 32402120

[ref41] Conca D. V.; Bano F.; Wirén J. von.; Scherrer L.; Svirelis J.; Thorsteinsson K.; Dahlin A.; Bally M. Variant-Specific Interactions at the Plasma Membrane: Heparan Sulfate’s Impact on SARS-CoV-2 Binding Kinetics bioRxiv 2024, p 2024-01 10.1101/2024.01.10.574981. PMC11883730 39976108

[ref42] Norling K.; Bernasconi V.; Agmo Hernández V.; Parveen N.; Edwards K.; Lycke N. Y.; Hoök F.; Bally M. Gel Phase 1,2-Distearoyl-Sn-Glycero-3-Phosphocholine-Based Liposomes Are Superior to Fluid Phase Liposomes at Augmenting Both Antigen Presentation on Major Histocompatibility Complex Class II and Costimulatory Molecule Display by Dendritic Cells in Vitro. ACS Infect. Dis. 2019, 5 (11), 1867–1878. 10.1021/acsinfecdis.9b00189. 31498993

[ref43] Ofir-Birin Y.; Abou karam P.; Rudik A.; Giladi T.; Porat Z.; Regev-Rudzki N. Monitoring Extracellular Vesicle Cargo Active Uptake by Imaging Flow Cytometry. Front. Immunol. 2018, 9, 1011 10.3389/fimmu.2018.01011. 29881375 PMC5976745

[ref44] Zuba-Surma E. K.; Kucia M.; Abdel-latif A. A.; Lillard J. W.; Ratajczak M. Z. The ImageStream System: A Key Step to a New Era in Imaging. Folia Histochem. Cytobiol. 2007, 45 (4), 279–290. 18165167

[ref45] Stegmann T.; Wey J.; Bartoldus I.; Schoen P.; Bron R.; Ortiz A.; Nieva J. L.; Wilschut J. Evaluation of Viral Membrane Fusion Assays. Comparison of the Octadecylrhodamine Dequenching Assay with the Pyrene Excimer Assay. Biochemistry 1993, 32 (42), 11330–11337. 10.1021/bi00093a009. 8218197

[ref46] MacDonald R. I. Characteristics of Self-Quenching of the Fluorescence of Lipid-Conjugated Rhodamine in Membranes. J. Biol. Chem. 1990, 265 (23), 13533–13539. 10.1016/S0021-9258(18)77380-8. 2380172

[ref47] Arbeloa F. L.; Ojeda P. R.; Arbeloa I. L. Flourescence Self-Quenching of the Molecular Forms of Rhodamine B in Aqueous and Ethanolic Solutions. J. Lumin. 1989, 44 (1–2), 105–112. 10.1016/0022-2313(89)90027-6.

[ref48] Zhao W.; Fogg D. K.; Kaplan M. J. A Novel Image-Based Quantitative Method for the Characterization of NETosis. J. Immunol. Methods 2015, 423, 104–110. 10.1016/j.jim.2015.04.027. 26003624 PMC4522197

[ref49] Lindner B.; Martin E.; Steininger M.; Bundalo A.; Lenter M.; Zuber J.; Schuler M. A Genome-Wide CRISPR/Cas9 Screen to Identify Phagocytosis Modulators in Monocytic THP-1 Cells. Sci. Rep. 2021, 11 (1), 12973 10.1038/s41598-021-92332-7. 34155263 PMC8217514

[ref50] Kurynina A. V.; Erokhina M. V.; Makarevich O. A.; Sysoeva V. Y.; Lepekha L. N.; Kuznetsov S. A.; Onishchenko G. E. Plasticity of Human THP–1 Cell Phagocytic Activity during Macrophagic Differentiation. Biochemistry 2018, 83 (3), 200–214. 10.1134/S0006297918030021. 29625541

[ref51] Mao Y.; Finnemann S. C. Regulation of Phagocytosis by Rho GTPases. Small GTPases 2015, 6 (2), 89–99. 10.4161/21541248.2014.989785. 25941749 PMC4601285

[ref52] Dean P.; Heunis T.; Härtlova A.; Trost M. Regulation of Phagosome Functions by Post-Translational Modifications: A New Paradigm. Curr. Opin. Chem. Biol. 2019, 48, 73–80. 10.1016/j.cbpa.2018.11.001. 30481638

[ref53] Cockram T. O. J.; Dundee J. M.; Popescu A. S.; Brown G. C. The Phagocytic Code Regulating Phagocytosis of Mammalian Cells. Front. Immunol. 2021, 12, 629979 10.3389/fimmu.2021.629979. 34177884 PMC8220072

[ref54] Richards D. M.; Endres R. G. The Mechanism of Phagocytosis: Two Stages of Engulfment. Biophys. J. 2014, 107 (7), 1542–1553. 10.1016/j.bpj.2014.07.070. 25296306 PMC4190621

[ref55] McKelvey K. J.; Powell K. L.; Ashton A. W.; Morris J. M.; McCracken S. A. Exosomes: Mechanisms of Uptake. J. Circ. Biomarkers 2015, 4, 7 10.5772/61186. PMC5572985 28936243

[ref56] Chang M. Y.; Brune J. E.; Black M.; Altemeier W. A.; Frevert C. W. Multicompartmental Analysis of the Murine Pulmonary Immune Response by Spectral Flow Cytometry. Am. J. Physiol. Lung Cell. Mol. Physiol. 2023, 325 (4), L518–L535. 10.1152/ajplung.00317.2022. 37581225 PMC10639014

[ref57] Croce A. C.; Bottiroli G. Autofluorescence Spectroscopy and Imaging: A Tool for Biomedical Research and Diagnosis. Eur. J. Histochem. 2014, 58 (4), 320–337. 10.4081/ejh.2014.2461. PMC4289852 25578980

[ref58] Thottacherry J. J.; Sathe M.; Prabhakara C.; Mayor S. Spoilt for Choice: Diverse Endocytic Pathways Function at the Cell Surface. Annu. Rev. Cell Dev. Biol. 2019, 35 (1), 55–84. 10.1146/annurev-cellbio-100617-062710. 31283376 PMC6917507

[ref59] Andronico L. A.; Jiang Y.; Carannante V.; Iskrak S.; Sandoz P. A.; Mikes J.; Klymchenko A.; Buggert M.; Österborg A.; Önfelt B.; Brodin P.; Sezgin E. High-Throughput Analysis of Membrane Fluidity Unveils a Hidden Dimension in Immune Cell States bioRxiv 2024, p 2024-01 10.1101/2024.01.15.575649.

[ref60] Schneider J.; Dufrêne Y. F.; Barger W. R.; Lee G. U. Atomic Force Microscope Image Contrast Mechanisms on Supported Lipid Bilayers. Biophys. J. 2000, 79 (2), 1107–1118. 10.1016/S0006-3495(00)76364-8. 10920040 PMC1301006

[ref61] Garcia-Manyes S.; Sanz F. Nanomechanics of Lipid Bilayers by Force Spectroscopy with AFM: A Perspective. Biochim. Biophys. Acta, Biomembr. 2010, 1798 (4), 741–749. 10.1016/j.bbamem.2009.12.019. 20044974

[ref62] Dufrêne Y. F.; Boland T.; Schneider J. W.; Barger W. R.; Lee G. U. Characterization of the Physical Properties of Model Biomembranes at the Nanometer Scale with the Atomic Force Microscope. Faraday Discuss. 1999, 111 (0), 79–94. 10.1039/a807637e. 10822601

[ref63] Raucher D.; Sheetz M. P. Membrane Expansion Increases Endocytosis Rate during Mitosis. J. Cell Biol. 1999, 144 (3), 497–506. 10.1083/jcb.144.3.497. 9971744 PMC2132908

[ref64] Djakbarova U.; Madraki Y.; Chan E. T.; Kural C. Dynamic Interplay between Cell Membrane Tension and Clathrin-Mediated Endocytosis. Biol. Cell 2021, 113 (8), 344–373. 10.1111/boc.202000110. 33788963 PMC8898183

[ref65] Rodal S. K.; Skretting G.; Garred Ø.; Vilhardt F.; Van Deurs B.; Sandvig K. Extraction of Cholesterol with Methyl-β-Cyclodextrin Perturbs Formation of Clathrin-Coated Endocytic Vesicles. Mol. Biol. Cell 1999, 10 (4), 961–974. 10.1091/mbc.10.4.961. 10198050 PMC25220

[ref66] Hissa B.; Pontes B.; Roma P. M. S.; Alves A. P.; Rocha C. D.; Valverde T. M.; Aguiar P. H. N.; Almeida F. P.; Guimarães A. J.; Guatimosim C.; Silva A. M.; Fernandes M. C.; Andrews N. W.; Viana N. B.; Mesquita O. N.; Agero U.; Andrade L. O. Membrane Cholesterol Removal Changes Mechanical Properties of Cells and Induces Secretion of a Specific Pool of Lysosomes. PLoS One 2013, 8 (12), e82988 10.1371/journal.pone.0082988. 24376622 PMC3869752

[ref67] Biswas A.; Kashyap P.; Datta S.; Sengupta T.; Sinha B. Cholesterol Depletion by MβCD Enhances Cell Membrane Tension and Its Variations-Reducing Integrity. Biophys. J. 2019, 116 (8), 1456–1468. 10.1016/j.bpj.2019.03.016. 30979551 PMC6486507

[ref68] Yáñez-Mó M.; Siljander P.R.; Andreu Z.; Zavec A.B.; Borràs F.E.; Buzas E.I.; Buzas K.; Casal E.; Cappello F.; Carvalho J.; et al. Biological properties of extracellular vesicles and their physiological functions. J Extracell Vesicles 2015, 14 (4), 27066 10.3402/jev.v4.27066. PMC4433489 25979354

[ref69] Owen D. M. Methods in Membrane Lipids, 2nd ed.; Humana Press, 2015; Vol. 1232, pp 1–327.

[ref70] Park D.; Don A. S.; Massamiri T.; Karwa A.; Warner B.; MacDonald J.; Hemenway C.; Naik A.; Kuan K. T.; Dilda P. J.; Wong J. W. H.; Camphausen K.; Chinen L.; Dyszlewski M.; Hogg P. J. Noninvasive Imaging of Cell Death Using an Hsp90 Ligand. J. Am. Chem. Soc. 2011, 133 (9), 2832–2835. 10.1021/ja110226y. 21322555 PMC7371243

[ref71] Krylova S. V.; Feng D. The Machinery of Exosomes: Biogenesis, Release, and Uptake. Int. J. Mol. Sci. 2023, 24 (2), 1337 10.3390/ijms24021337. 36674857 PMC9865891

[ref72] Rai A. K.; Johnson P. J. Trichomonas Vaginalis Extracellular Vesicles Are Internalized by Host Cells Using Proteoglycans and Caveolin-Dependent Endocytosis. Proc. Natl. Acad. Sci. U.S.A. 2019, 116 (43), 21354–21360. 10.1073/pnas.1912356116. 31601738 PMC6815132

[ref73] Grant B. D.; Donaldson J. G. Pathways and Mechanisms of Endocytic Recycling. Nat. Rev. Mol. Cell Biol. 2009, 10 (9), 597–608. 10.1038/nrm2755. 19696797 PMC3038567

[ref74] Cocucci E.; Aguet F.; Boulant S.; Kirchhausen T. The First Five Seconds in the Life of a Clathrin-Coated Pit. Cell 2012, 150 (3), 495–507. 10.1016/j.cell.2012.05.047. 22863004 PMC3413093

[ref75] François-Martin C.; Pincet F. Actual Fusion Efficiency in the Lipid Mixing Assay - Comparison between Nanodiscs and Liposomes. Sci. Rep. 2017, 7 (1), 43860 10.1038/srep43860. 28266607 PMC5339690

[ref76] Cho Y. Y.; Kwon O. H.; Chung S. Preferred Endocytosis of Amyloid Precursor Protein from Cholesterol-Enriched Lipid Raft Microdomains. Molecules 2020, 25 (23), 5490 10.3390/molecules25235490. 33255194 PMC7727664

[ref77] Watts C.; Marsh M. Endocytosis: What Goes in and How?. J. Cell Sci. 1992, 103 (1), 1–8. 10.1242/JCS.103.1.1A. 1429899

[ref78] Morandi M. I.; Busko P.; Ozer-Partuk E.; Khan S.; Zarfati G.; Elbaz-Alon Y.; Abou Karam P.; Napso Shogan T.; Ginini L.; Gil Z.; Regev-Rudzki N.; Avinoam O. Extracellular Vesicle Fusion Visualized by Cryo-Electron Microscopy. PNAS Nexus 2022, 1 (4), pgac156 10.1093/pnasnexus/pgac156. 36714848 PMC9802263

[ref79] Ben Ami Pilo H.; Khan Khilji S.; Lühle J.; Biskup K.; Levy Gal B.; Rosenhek Goldian I.; Alfandari D.; Revach O.; Kiper E.; Morandi M. I.; Rotkopf R.; Porat Z.; Blanchard V.; Seeberger P. H.; Regev-Rudzki N.; Moscovitz O. Sialylated N -glycans Mediate Monocyte Uptake of Extracellular Vesicles Secreted from Plasmodium Falciparum -infected Red Blood Cells. J. Extracell. Biol. 2022, 1 (2), e33 10.1002/jex2.33. 38938665 PMC11080922

[ref80] Näslund T. I.; Paquin-Proulx D.; Paredes P. T.; Vallhov H.; Sandberg J. K.; Gabrielsson S. Exosomes from Breast Milk Inhibit HIV-1 Infection of Dendritic Cells and Subsequent Viral Transfer to CD4+ T Cells. Aids 2014, 28 (2), 171–180. 10.1097/QAD.0000000000000159. 24413309

[ref81] Rana S.; Yue S.; Stadel D.; Zöller M. Toward Tailored Exosomes: The Exosomal Tetraspanin Web Contributes to Target Cell Selection. Int. J. Biochem. Cell Biol. 2012, 44 (9), 1574–1584. 10.1016/j.biocel.2012.06.018. 22728313

[ref82] Zech D.; Rana S.; Büchler M. W.; Zöller M. Tumor-Exosomes and Leukocyte Activation: An Ambivalent Crosstalk. Cell Commun. Signaling 2012, 10, 37 10.1186/1478-811X-10-37. PMC3519567 23190502

[ref83] Groza R.; Schmidt K. V.; Müller P. M.; Ronchi P.; Schlack-Leigers C.; Neu U.; Puchkov D.; Dimova R.; Matthaeus C.; Taraska J.; Weikl T. R.; Ewers H. Adhesion Energy Controls Lipid Binding-Mediated Endocytosis. Nat. Commun. 2024, 15 (1), 2767 10.1038/s41467-024-47109-7. 38553473 PMC10980822

[ref84] Martin-Jaular L.; Nakayasu E. S.; Ferrer M.; Almeida I. C.; del Portillo H. A. Exosomes from Plasmodium Yoelii-Infected Reticulocytes Protect Mice from Lethal Infections. PLoS One 2011, 6 (10), e26588 10.1371/journal.pone.0026588. 22046311 PMC3202549

[ref85] Trager W.; Jensen J. B. Human Malaria Parasites in Continuous Culture. J. Parasitol. 2005, 91 (3), 484–486. 10.1645/0022-3395(2005)091[0484:HMPICC]2.0.CO;2. 16108535

[ref86] Unterholzner L.; Keating S. E.; Baran M.; Horan K. A.; Jensen S. B.; Sharma S.; Sirois C. M.; Jin T.; Latz E.; Xiao T. S.; Fitzgerald K. A.; Paludan S. R.; Bowie A. G. IFI16 Is an Innate Immune Sensor for Intracellular DNA. Nat. Immunol. 2010, 11 (11), 997–1004. 10.1038/ni.1932. 20890285 PMC3142795

[ref87] Cladera J.; Martin I.; O’Shea P. The Fusion Domain of HIV Gp41 Interacts Specifically with Heparan Sulfate on the T-Lymphocyte Cell Surface. EMBO J. 2001, 20 (1–2), 19–26. 10.1093/emboj/20.1.19. 11226151 PMC140179

[ref88] Klug Y. A.; Schwarzer R.; Rotem E.; Charni M.; Nudelman A.; Gramatica A.; Zarmi B.; Rotter V.; Shai Y. The HIV Gp41 Fusion Protein Inhibits T-Cell Activation through the Lentiviral Lytic Peptide 2 Motif. Biochemistry 2019, 58 (6), 818–832. 10.1021/acs.biochem.8b01175. 30602116

[ref89] Coleman B. M.; Hanssen E.; Lawson V. A.; Hill A. F. Prion-Infected Cells Regulate the Release of Exosomes with Distinct Ultrastructural Features. FASEB J. 2012, 26 (10), 4160–4173. 10.1096/fj.11-202077. 22767229

[ref90] Gerstle Z.; Desai R.; Veatch S. L. Giant Plasma Membrane Vesicles: An Experimental Tool for Probing the Effects of Drugs and Other Conditions on Membrane Domain Stability. Methods Enzymol. 2018, 603, 129–150. 10.1016/bs.mie.2018.02.007. 29673522 PMC6070695

[ref91] Filipe V.; Hawe A.; Jiskoot W. Critical Evaluation of Nanoparticle Tracking Analysis (NTA) by NanoSight for the Measurement of Nanoparticles and Protein Aggregates. Pharm. Res. 2010, 27 (5), 796–810. 10.1007/s11095-010-0073-2. 20204471 PMC2852530

[ref92] Fendl B.; Weiss R.; Fischer M. B.; Spittler A.; Weber V. Characterization of Extracellular Vesicles in Whole Blood: Influence of Pre-Analytical Parameters and Visualization of Vesicle-Cell Interactions Using Imaging Flow Cytometry. Biochem. Biophys. Res. Commun. 2016, 478 (1), 168–173. 10.1016/j.bbrc.2016.07.073. 27444383

[ref93] Nečas D.; Klapetek P. Gwyddion: An Open-Source Software for SPM Data Analysis. Open Phys. 2012, 10 (1), 181–188. 10.2478/s11534-011-0096-2.

[ref94] Virtanen P.; Gommers R.; Oliphant T. E.; Haberland M.; Reddy T.; Cournapeau D.; Burovski E.; Peterson P.; Weckesser W.; Bright J.; van der Walt S. J.; Brett M.; Wilson J.; Millman K. J.; Mayorov N.; Nelson A. R. J.; Jones E.; Kern R.; Larson E.; Carey C. J.; Polat İ.; Feng Y.; Moore E. W.; VanderPlas J.; Laxalde D.; Perktold J.; Cimrman R.; Henriksen I.; Quintero E. A.; Harris C. R.; Archibald A. M.; Ribeiro A. H.; Pedregosa F.; van Mulbregt P.; Vijaykumar A.; Bardelli A. Pietro.; Rothberg A.; Hilboll A.; Kloeckner A.; Scopatz A.; Lee A.; Rokem A.; Woods C. N.; Fulton C.; Masson C.; Häggström C.; Fitzgerald C.; Nicholson D. A.; Hagen D. R.; Pasechnik D. V.; Olivetti E.; Martin E.; Wieser E.; Silva F.; Lenders F.; Wilhelm F.; Young G.; Price G. A.; Ingold G. L.; Allen G. E.; Lee G. R.; Audren H.; Probst I.; Dietrich J. P.; Silterra J.; Webber J. T.; Slavič J.; Nothman J.; Buchner J.; Kulick J.; Schönberger J. L.; de Miranda Cardoso J. V.; Reimer J.; Harrington J.; Rodríguez J. L. C.; Nunez-Iglesias J.; Kuczynski J.; Tritz K.; Thoma M.; Newville M.; Kümmerer M.; Bolingbroke M.; Tartre M.; Pak M.; Smith N. J.; Nowaczyk N.; Shebanov N.; Pavlyk O.; Brodtkorb P. A.; Lee P.; McGibbon R. T.; Feldbauer R.; Lewis S.; Tygier S.; Sievert S.; Vigna S.; Peterson S.; More S.; Pudlik T.; Oshima T.; Pingel T. J.; Robitaille T. P.; Spura T.; Jones T. R.; Cera T.; Leslie T.; Zito T.; Krauss T.; Upadhyay U.; Halchenko Y. O.; Vázquez-Baeza Y. SciPy 1.0: Fundamental Algorithms for Scientific Computing in Python. Nat. Methods 2020, 17 (3), 261–272. 10.1038/s41592-019-0686-2. 32015543 PMC7056644

[ref95] Pedregosa F.; Michel V.; Grisel O.; Blondel M.; Prettenhofer P.; Weiss R.; Vanderplas J.; Cournapeau D.; Pedregosa F.; Varoquaux G.; Gramfort A.; Thirion B.; Grisel O.; Dubourg V.; Passos A.; Brucher M. Scikit-Learn: Machine Learning in Python. J. Mach. Learn. Res. 2011, 12 (85), 2825–2830.

