# Supplementary material for: Host Immune Cell Membrane Deformability Governs the Uptake Route of Malaria-Derived Extracellular Vesicles
Source: ACS Nano. 2025 Mar 3;19(10):9760–78. doi: 10.1021/acsnano.4c07503 (PMC11924330; doi:10.1021/acsnano.4c07503)
Supplement: Supplementary file 1 — nn4c07503_si_001.pdf [file nn4c07503_si_001.pdf]

Supplementary Information

Host Immune Cell Membrane Deformability Governs the Uptake Route of Malaria-Derived Extracellular Vesicles

Daniel Alfandari[1], Irit Rosenhek-Goldian[2], Ewa Kozela[1], Reinat Nevo[1], Marcela Bahlsen Senprún[1], Anton Moisieiev[1], Noam Sogauker[1], Ido Azuri[3], Samuel Gelman[3], Edo Kiper[1], Daniel Ben Hur[1], Raviv Dharan[4], Raya Sorkin[4], Ziv Porat[5], Mattia I. Morandi[6,7*] and Neta Regev-Rudzki[1*]

[1]Department of Biomolecular Sciences, Faculty of Biochemistry, Weizmann Institute of Science, Rehovot, 7610001, Israel
[2]Department of Chemical Research Support, Weizmann Institute of Science, Rehovot, 7610001, Israel
[3]Bioinformatics Unit, Life Sciences Core Facilities, Weizmann Institute of Science, Rehovot, 7610001, Israel
[4]Raymond and Beverly Sackler Faculty of Exact Sciences, School of Chemistry, Tel Aviv University, Tel Aviv, 6997801, Israel
[5]Flow cytometry Unit, Life Sciences Core Facilities, Weizmann Institute of Science, Rehovot, 7610001, Israel
[6]Institute of Organic Chemistry and Biochemistry of the Czech Academy of Science, Prague, 160-00, Czech Republic
[7]IMol Polish Academy of Sciences, Warsaw, 02-247, Poland

***Corresponding authors**:

mattia.morandi@uochb.cas.cz and neta.regev-rudzki@weizmann.ac.il

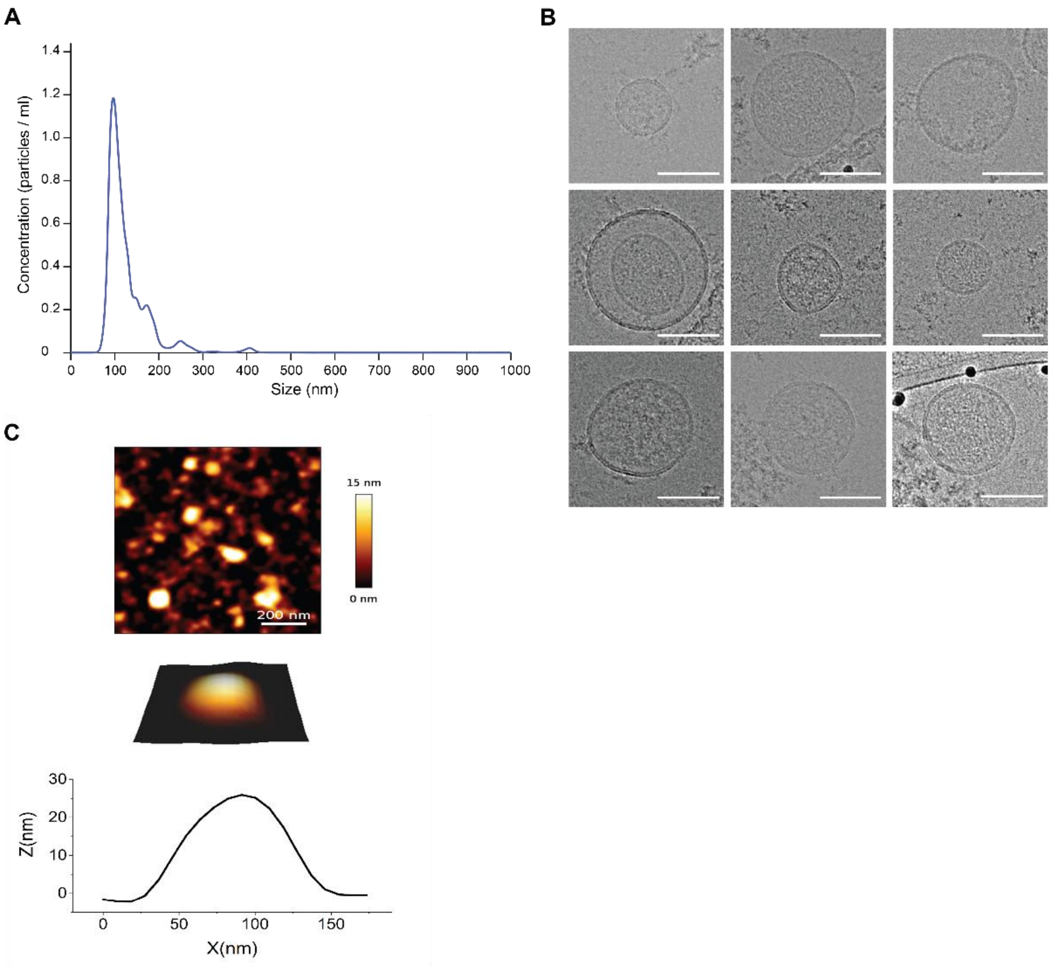

Figure S1. ***Pf*-derived EV characterization**. **(A)** Representative nanoparticle tracking analysis of *Pf*-derived EVs plotting particles per ml versus diameter. Average diameter was 96 nm. The EV concentration was approximately 10^{12} particles/ml. **(B)** Representative cryo-electron microscopy images of *Pf*-derived EVs isolated using OptPrep density gradient. Intact bilayer morphology was observed. Scale bars: 100 nm. **(C)** Representative 2D and 3D atomic force microscopy images of *Pf*-derived EVs.

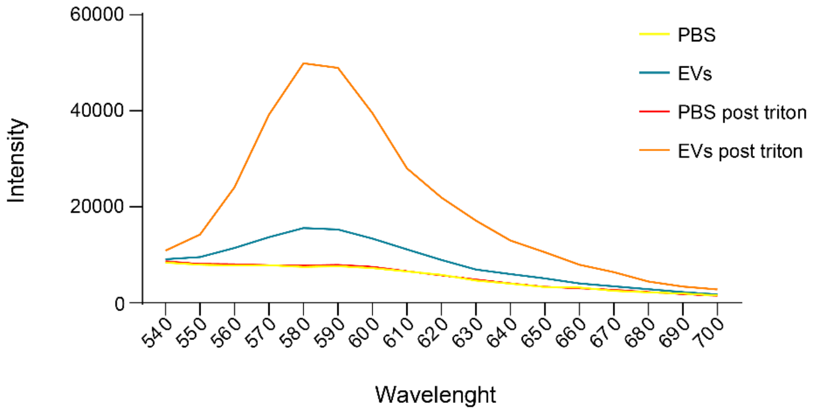

Figure S2. R18-labeled EVs show self-quenching behavior. Measurements were performed using an Infinite M Plex multimode plate reader (TECAN) in a 96-well plate. *Pf*-derived EVs were labeled with R18, and fluorescent intensity was measured with an excitation wavelength of 505 nm and emission wavelengths of 540 to 700 nm. After addition of 10% Triton X-100 to disrupt EVs, fluorescence intensity was measured again.

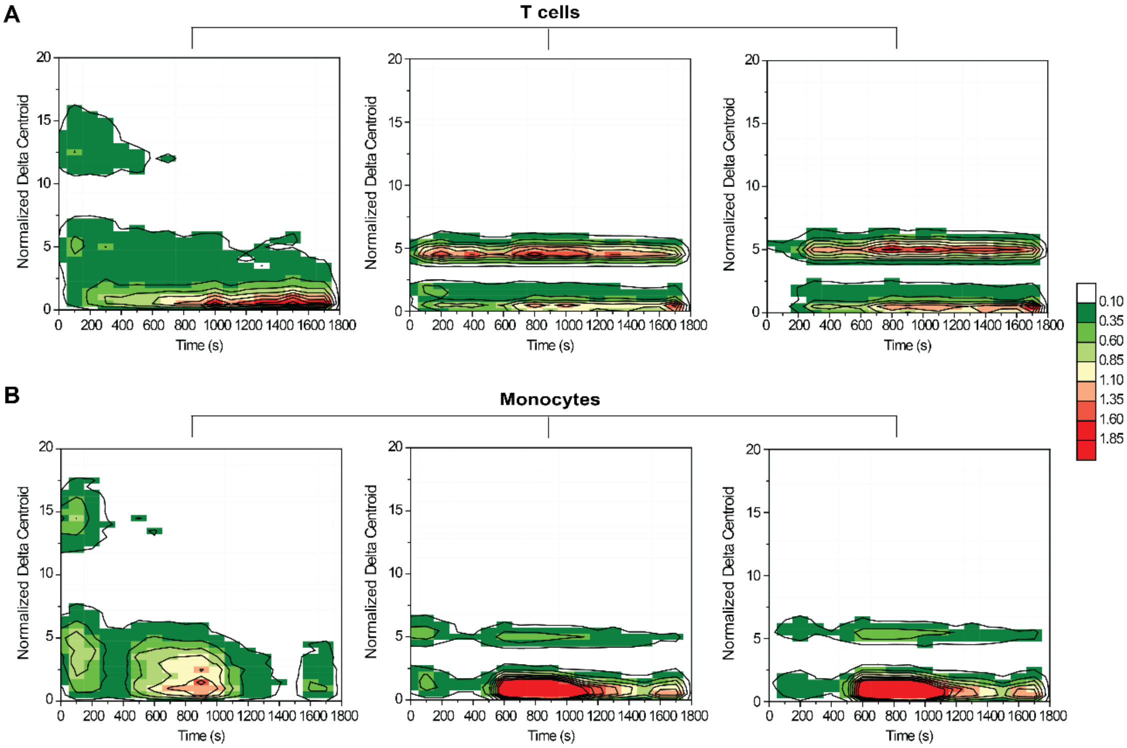

Figure S3. R18 dye alone does not show the spatial fluorescence distribution observed with R18-labeled EVs. (A) Heat maps of cellular R18 Δ^{norm}_{xy} relative frequency after incubation of R18 dye with T cells. **(B)** Heat maps of cellular R18 Δ^{norm}_{xy} relative frequency after incubation of R18 dye with monocytes. Data are from 3 independent biological samples.

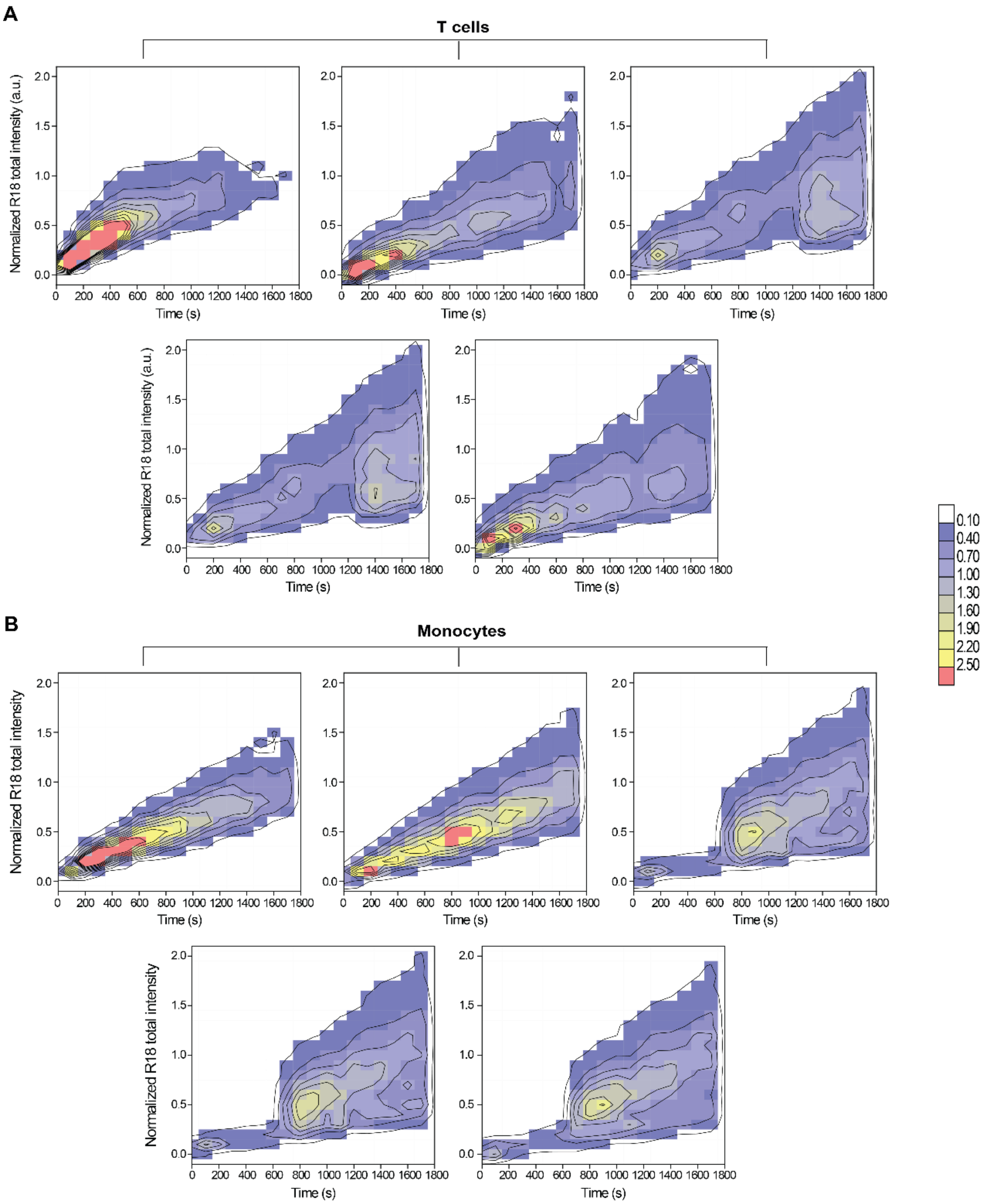
A
T cells
Normalized R18 total intensity (a.u.)
Time (s)
0 200 400 600 800 1000 1200 1400 1600 1800
0.0 0.5 1.0 1.5 2.0
0.10
0.40
0.70
1.00
1.30
1.60
1.90
2.20
2.50
B
Monocytes
Normalized R18 total intensity
Time (s)

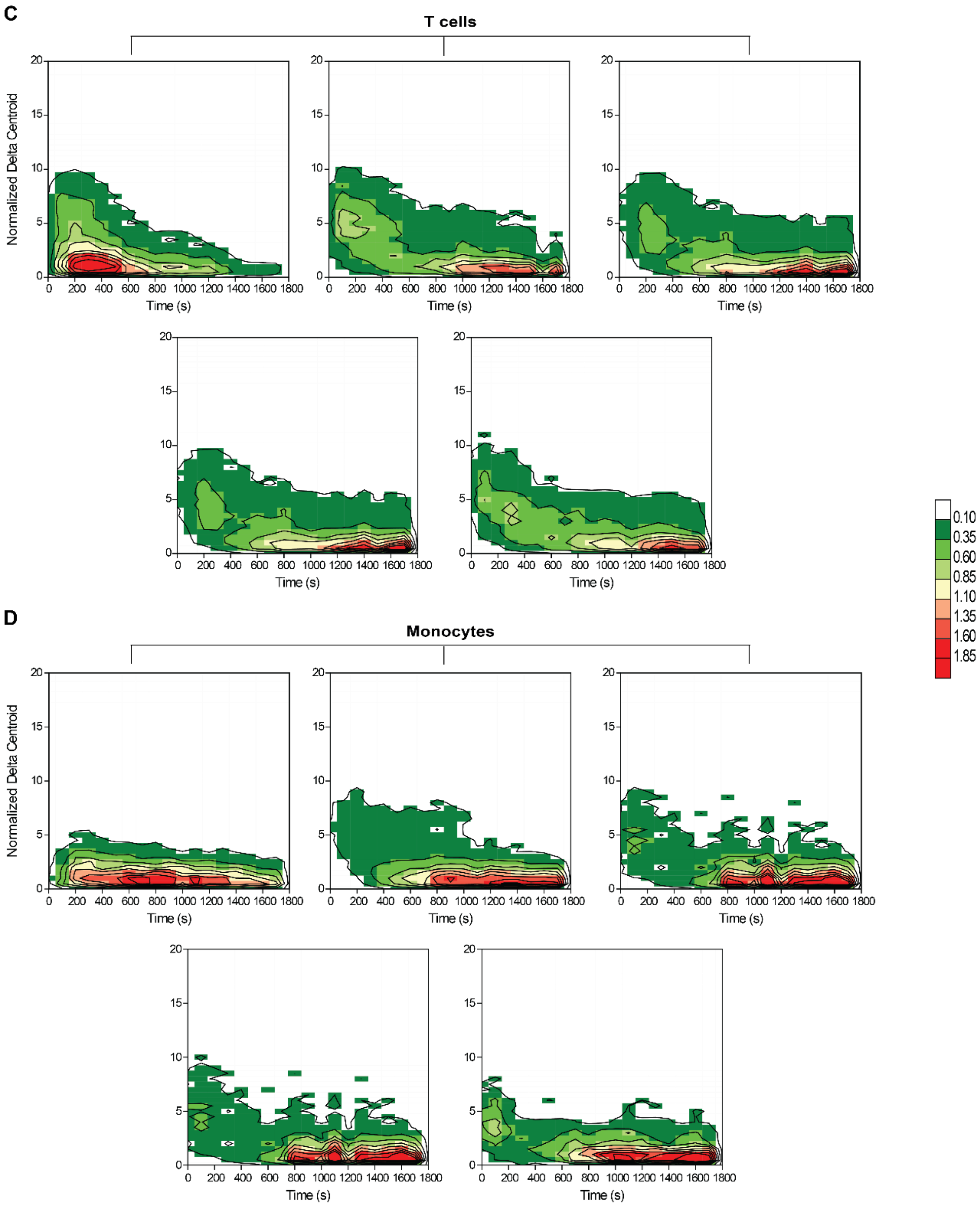

Figure S4. Data from technical repeats of analyses of R18 total intensity and intensity spatial distribution. (A) Heat maps of cellular R18 total intensity as a function of time of incubation of R18-labeled EVs with T cells. **(B)** Heat maps of cellular R18 total intensity as a function of time of incubation of R18-labeled EVs with monocytes. **(C)** Heat maps of cellular R18 Δ^{norm}_{xy} as a function of time of incubation of R18-labeled EVs with T cells. **(D)** Heat maps of cellular R18 Δ^{norm}_{xy} as a function of time of incubation of R18-labeled EVs with monocytes.

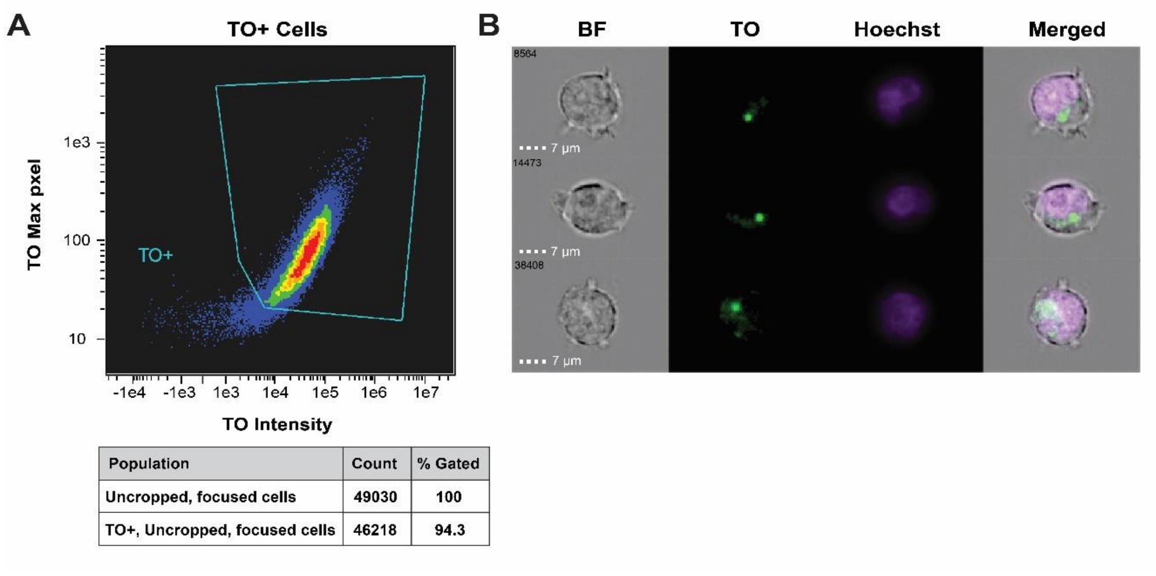

Population	Count	% Gated
Uncropped, focused cells	49030	100
TO+, Uncropped, focused cells	46218	94.3

Figure S5. Monitoring uptake of *Pf*-derived EVs into host T cells. *Pf*-derived EVs were labeled with TO, and their uptake into T cells was measured for 1 h. **(A)** Percentage of TO-positive cells after 30 min, gated by unlabeled EVs. Representative results from at least 3 independent experiments are shown. Host cells incubated with unstained EVs were used as controls. **(B)** Signal detected by IFC from three individual representative recipient cells of T cells, 30 min post uptake. BF, bright field.

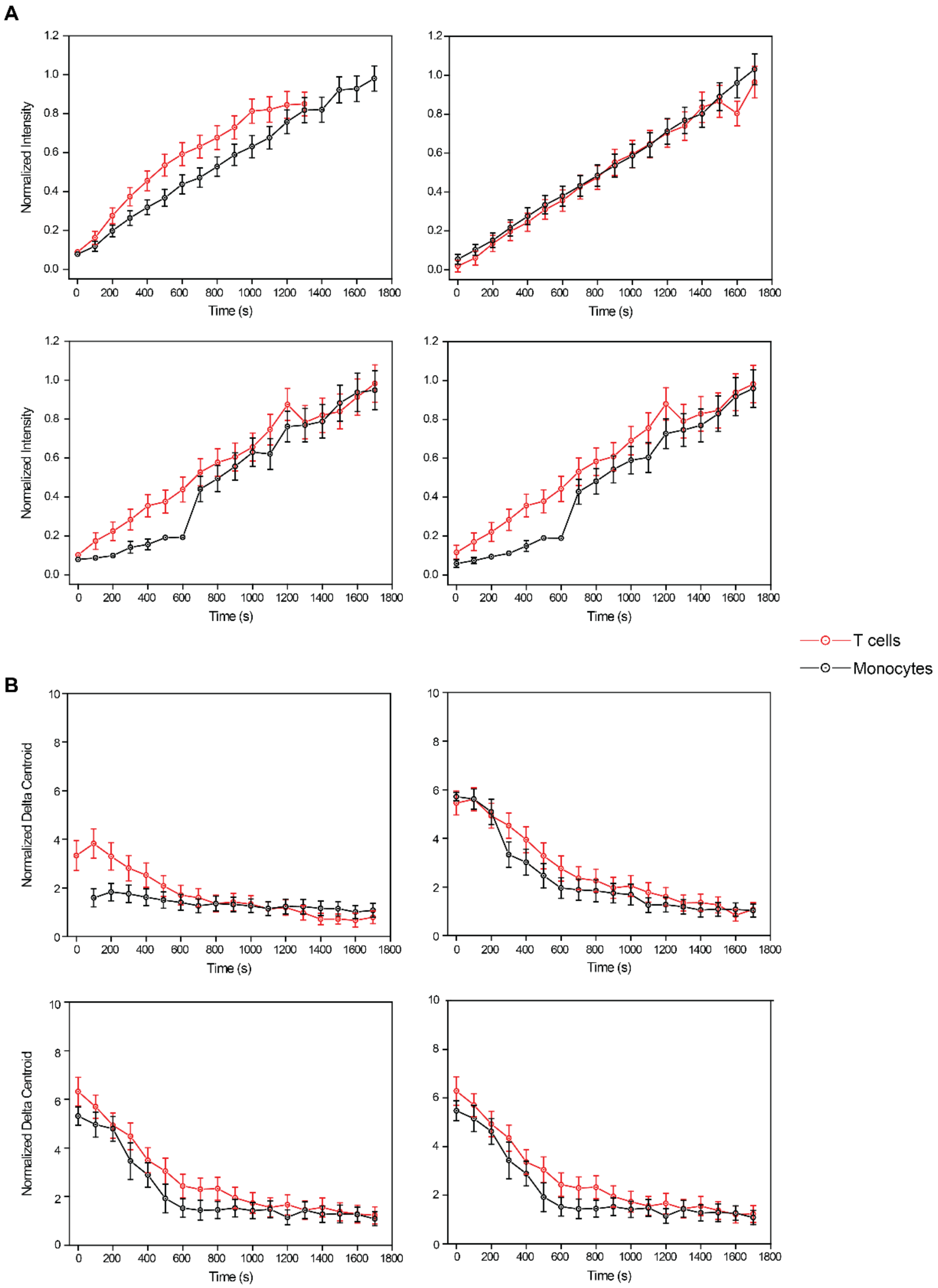

Figure S6. Data from technical repeats of R18 kinetic profiles. (A) Total R18 fluorescence intensity as a function of time of incubation of R18-labeled EVs with either T cells (red) or monocytes (black). **(B)** Cellular R18 Δ^{norm}_{xy} as a function of time of incubation of R18-labeled EVs with either T cells (red) or monocytes

(black). Each panel shows data from an independent biological repeat, with symbol representing the weighted average of the Δ^{norm}_{xy} at that specific time point and error bars representing weighted standard error.

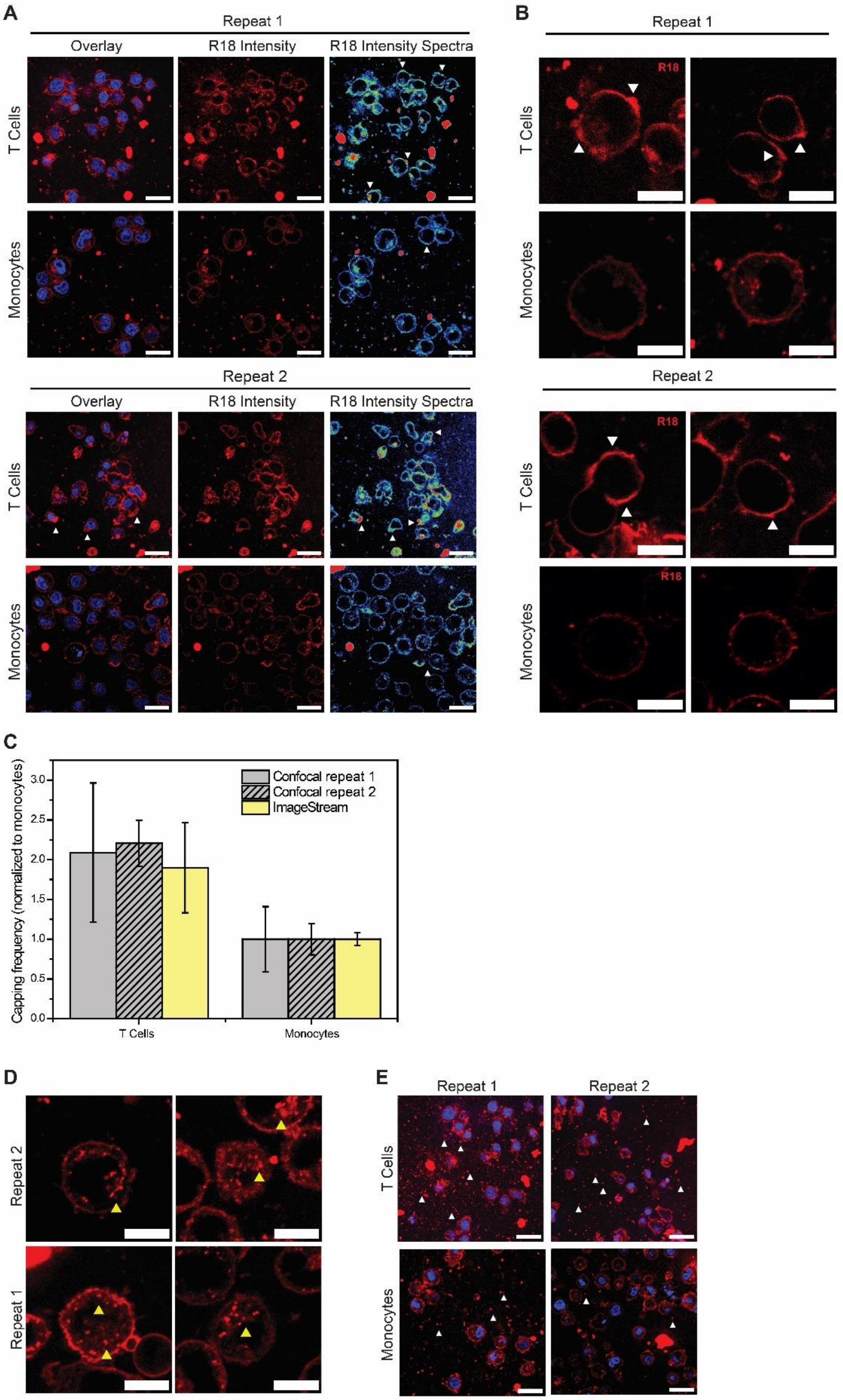
A
Repeat 1
Overlay
R18 Intensity
R18 Intensity Spectra
T Cells
Monocytes
Repeat 2
Overlay
R18 Intensity
R18 Intensity Spectra
T Cells
Monocytes
B
Repeat 1
R18
T Cells
Monocytes
Repeat 2
R18
T Cells
R18
Monocytes
C
Confocal repeat 1
Confocal repeat 2
ImageStream
Capping frequency (normalized to monocytes)
3.0
2.5
2.0
1.5
1.0
0.5
0.0
T Cells
Monocytes
D
Repeat 2
Repeat 1
E
Repeat 1
Repeat 2
T Cells
Monocytes

Figure S7. Confocal imaging of cells incubated with R18-labeled EVs showing different patterns of membrane fluorescent signal. (A) Representative confocal images of T cells and monocytes following 10-min incubation with R18-labeled EVs. Instances of polarized fluorescent signal (white arrows) are more common in T cells than monocytes. Images are presented as overlays of nuclear Hoechst staining and R18 fluorescence (left), R18 only signal (middle), and R18 signal presented as intensity spectra to better identify areas of higher fluorescence intensity (right). Scale bars: 20 µm. **(B)** Details for each cell type show high fluorescence intensity at the plasma membrane and the presence of membrane capping (white arrows) in T cells and low fluorescence intensity and uniform fluorescence distribution around the cellular membrane for monocytes. Scale bars: 10 µm. **(C)** Comparison of relative frequencies of T cells and monocytes displaying capping patterns in IFC and confocal images obtained from 542 cells (monocytes) and 661 cells (T Cells) from 2 biological replicates. Data are normalized to the frequency of monocytes. **(D)** Representative confocal microscopy images of monocytes incubated with R18-labeled EVs for 10 min, highlighting the presence of bright intracellular R18 puncta (yellow arrows) consistent with endocytic vesicles. Scale bars: 10 µm. **(E)** Representative confocal images showing R18-labeled EVs in solution (white arrows) in T cell and monocyte cultures. Scale bars: 20 µm. All confocal images are representative of 542 cells for monocytes and 661 cells for T Cells from 2 biological replicates.

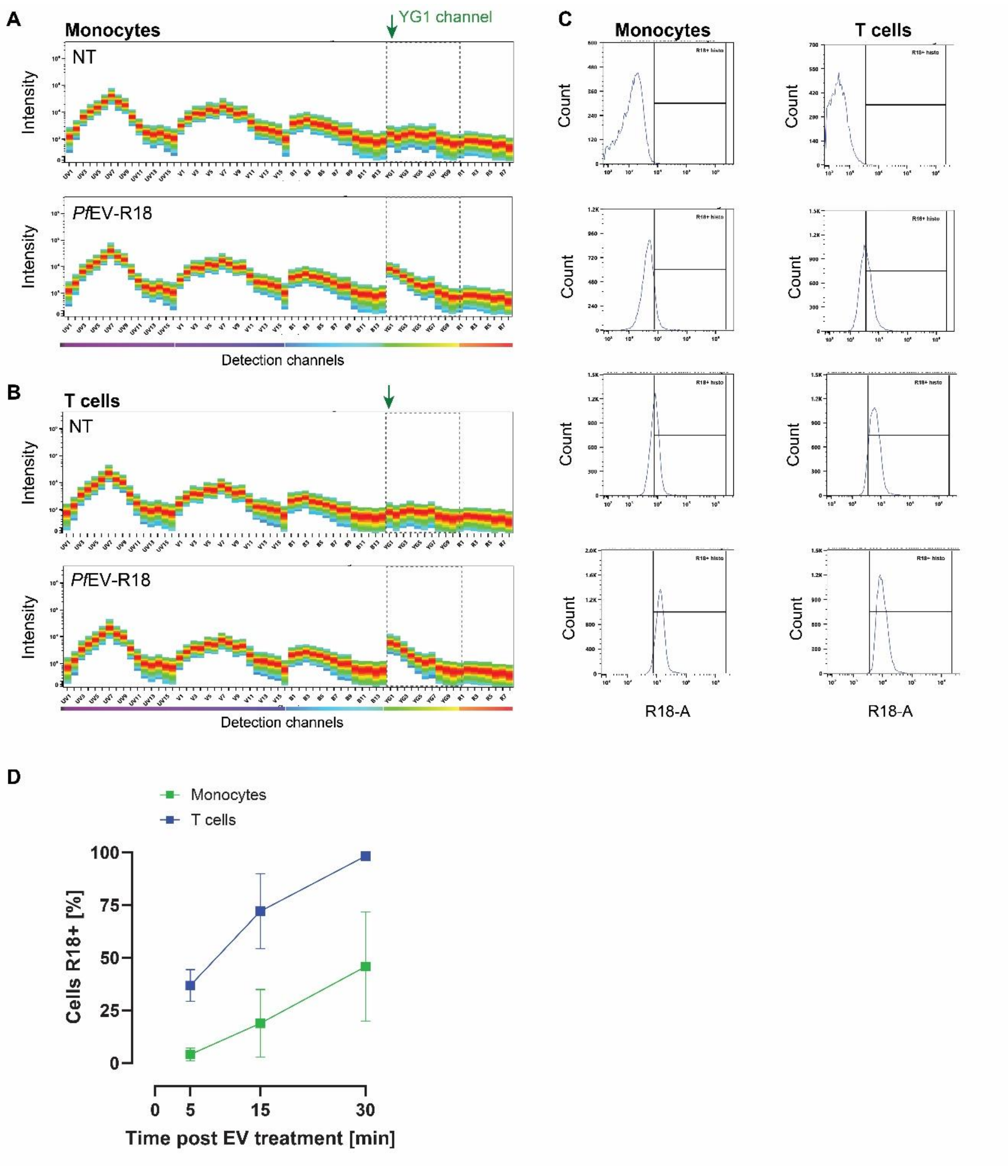

Figure S8. Kinetics of uptake of R18-labeled *Pf*-derived EVs into monocytes and T cells.

(A, B) Representative images of spectral signatures of **(A)** monocytes and of **(B)** T cells either not treated (NT) or treated for 30 min with R18-labeled *Pf*-derived EVs (10,000 EVs/cell; 5- and 15-min time points not shown). The areas within grey dashed lines show YG1-10 channels equivalent to R18-specific emission range and green arrow indicates YG1 peak channel. **(C)** Representative histograms of percentages of R18-positive monocytes and T cells in NT cells and in cells treated for 5, 15, or for 30 min with R18-labeled *Pf*-derived EVs (unmixing analysis). **(D)** Average percentages of R18-positive cells at 5, 15, and 30 min of incubation

with R18-labeled *Pf*-derived EVs (unmixing analysis was performed with autofluorescence subtraction; n = 3). Significance of difference between cells types (F(1,12)=16.2; p<0.01) and in the uptake kinetics (time points) of R18-labeled *Pf*-derived EVs (F(2,12=6.75); p<0.01) was evaluated with ANOVA.

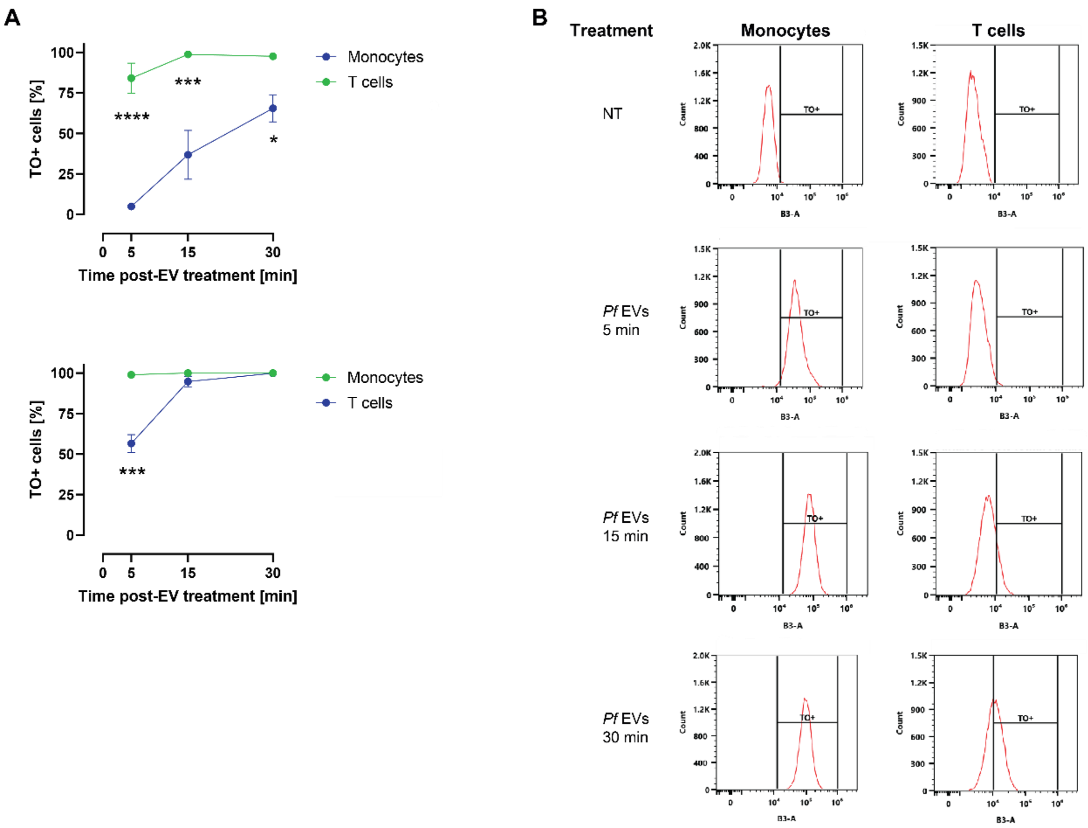

Figure S9. Monocytes and T cells exhibit distinct *Pf*-derived EV uptake kinetics (B3 channel intensity raw analysis). (A) Average percentages of TO^+ cells for each of the tested conditions (cell type, EV concentration/cell, time, n = 3-4) as a function of time. Statistical analysis upper graph (10,000 EVs/cell): ANOVA $F(5, 16) = 24.69$, $p<0.0001$ and in lower graph (30,000 EVs/cell) $F(5, 16) = 43.87$, $p<0.0001$, post hoc Šidak multiple comparisons test. *p<0.05, **p<0.01, ***p<0.001, ****p<0.0001 for monocytes *versus* T cells at the same time points. **(B)** Representative histogram plots for raw B3 peak channel intensities detected in TO^+ monocytes (left column) and in T cells (right column) obtained after incubation of the cells with TO-labeled EVs at 10,000 EVs/cell for times 5, 15, and 30 min.

A

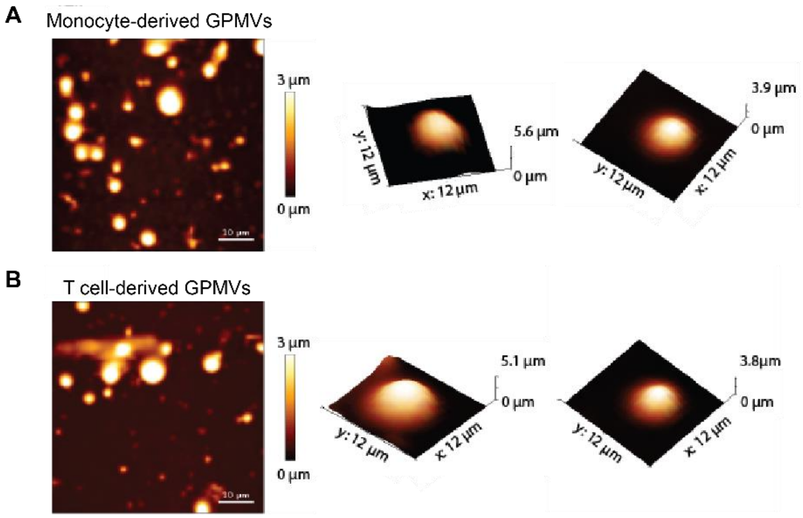

Figure S10. AFM characterization of GPMVs. Morphologies and sizes of GPMVs isolated from **(A)** monocytes and **(B)** T cells were evaluated using AFM.

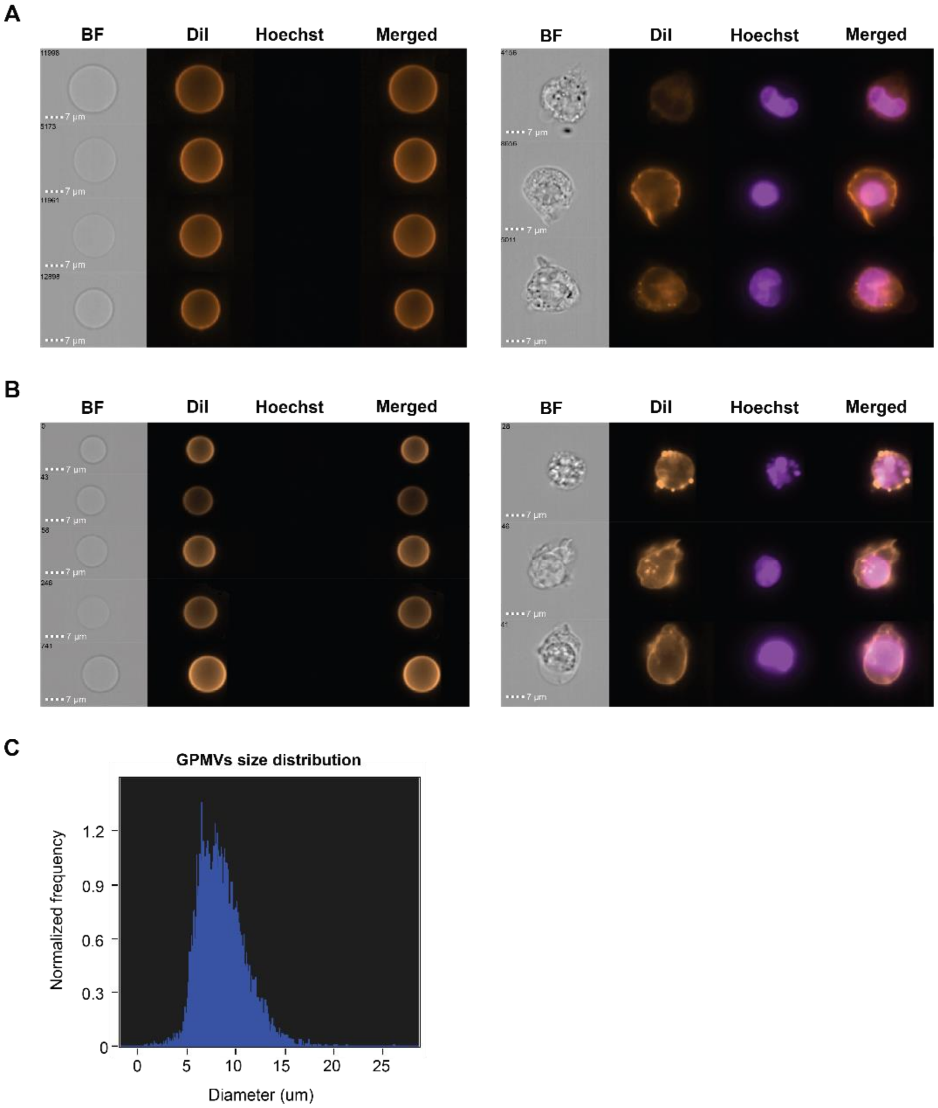

Figure S11. IFC characterization of GPMVs. GPMVs were isolated from monocytes labeled with DiI membrane dye and Hoechst nuclear stain. **(A, B)** Representative IFC images of **(A)** GPMVs and **(B)** monocytes. Brightfield (BF), DiI, Hoechst, and merged DiI and Hoechst signal are shown. **(C)** Diameter size range of GPMVs.

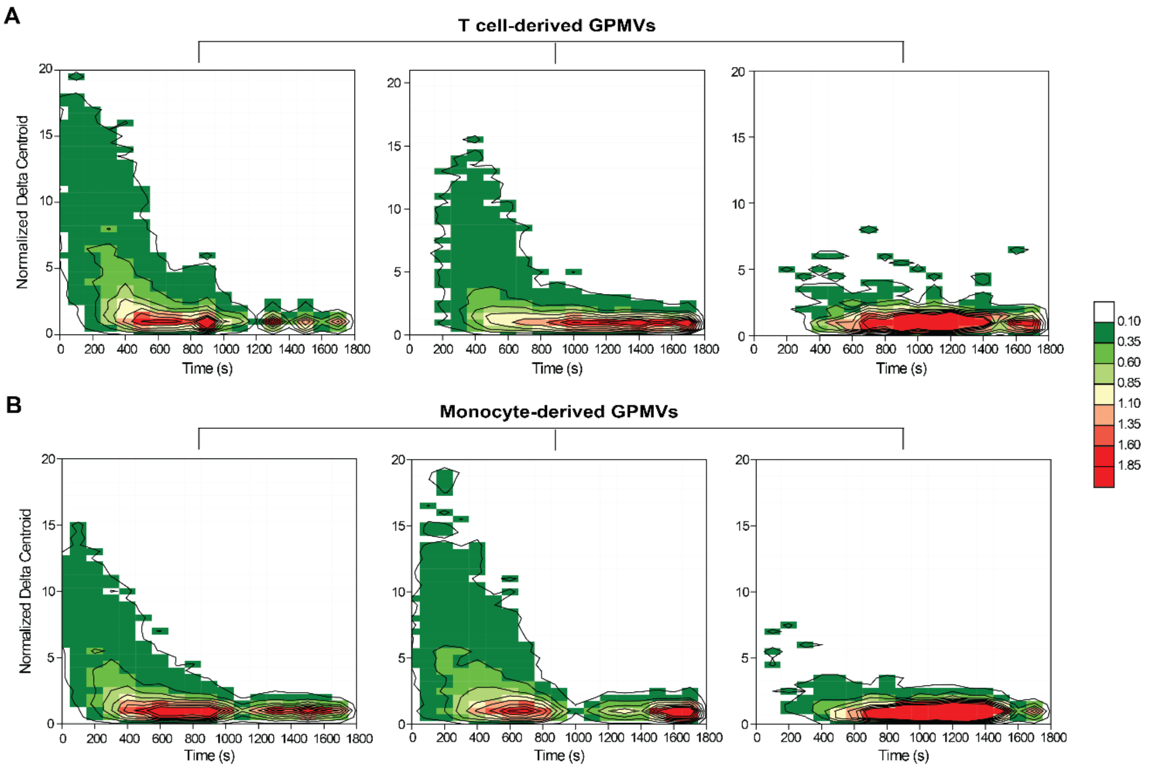

Figure S12. Data from technical repeats of analyses of R18 spatial distribution in GPMVs. (A) Heat maps of R18 Δ^{norm}_{xy} relative frequency after incubation of R18-labeled *Pf*-derived EVs with T cell-derived GPMVs. **(B)** Heat maps of R18 Δ^{norm}_{xy} relative frequency after incubation of R18-labeled EVs with monocyte-derived GPMVs. Each panel represents one from n = 3 independent biological repeats.

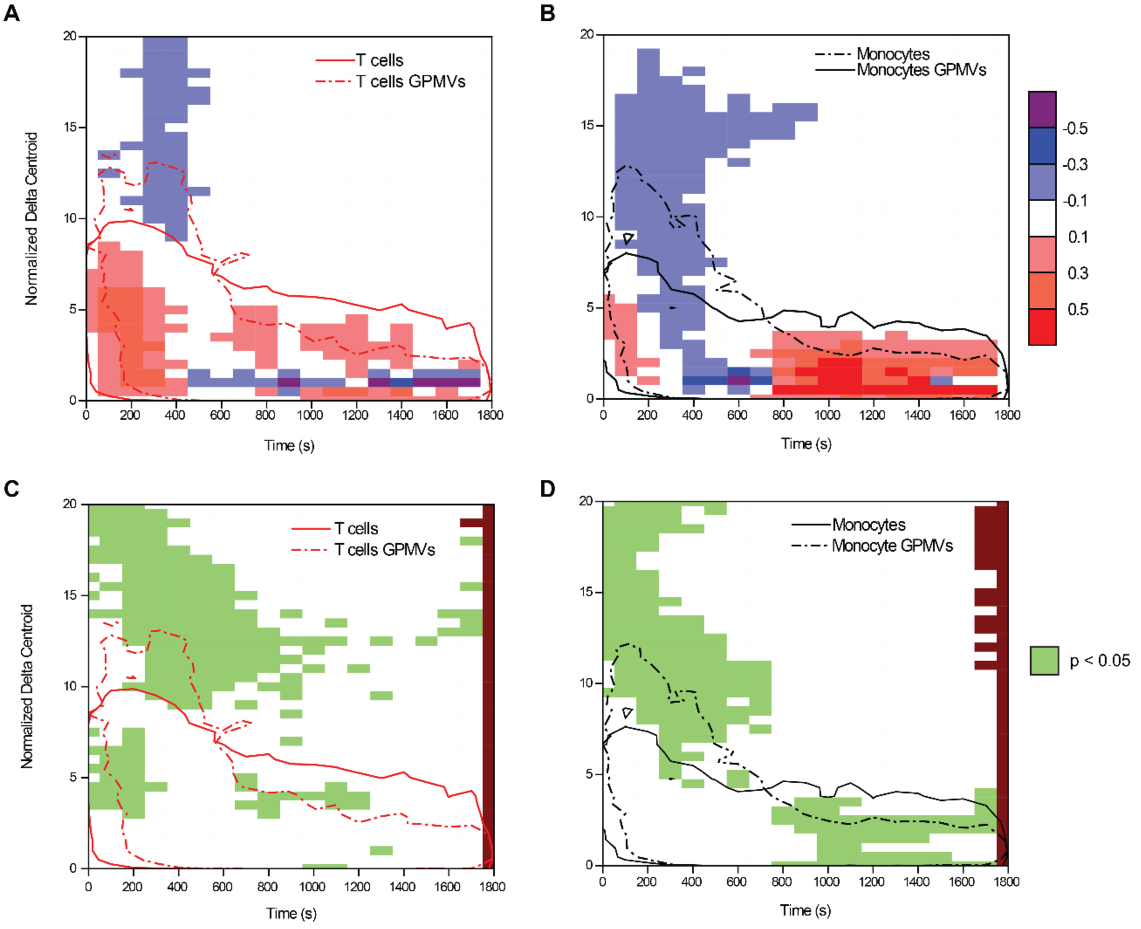

Figure S13. Comparison of characterization of EV uptake into intact cells and corresponding GPMVs. (A) Heat map of normalized Δxy values for intact T cells and T cell-derived GPMVs. **(B)** Heat map of normalized Δxy values for intact monocytes and monocyte-derived GPMVs. **(C)** Heat map of statistical significance of differences in Δxy values between intact T cells and T cell-derived GPMVs. **(D)** Heat map of statistical significance of differences in Δxy values intact monocytes and monocyte-derived GPMVs. In frequency-difference heat maps, blue areas indicate more events in GPMVs compared to intact T cells and red areas the opposite. In all panels, outlines indicate the isofrequency region at a relative frequency of 0.1% for either intact T cells (red line), intact monocytes (black line), T cell-derived GPMVs (red dashed line), and monocyte-derived GPMVs (black dashed line). Statistical significance was calculated using one-way ANOVA; green indicates statistical significance ($p < 0.05$), white indicates no significance (n.s.), and brown indicates statistical test not applicable. Data shown for cells were obtained from 5 independent biological repeats shown in Figures 2 and 3, and data shown for GPMVs were obtained from 3 biological repeats.

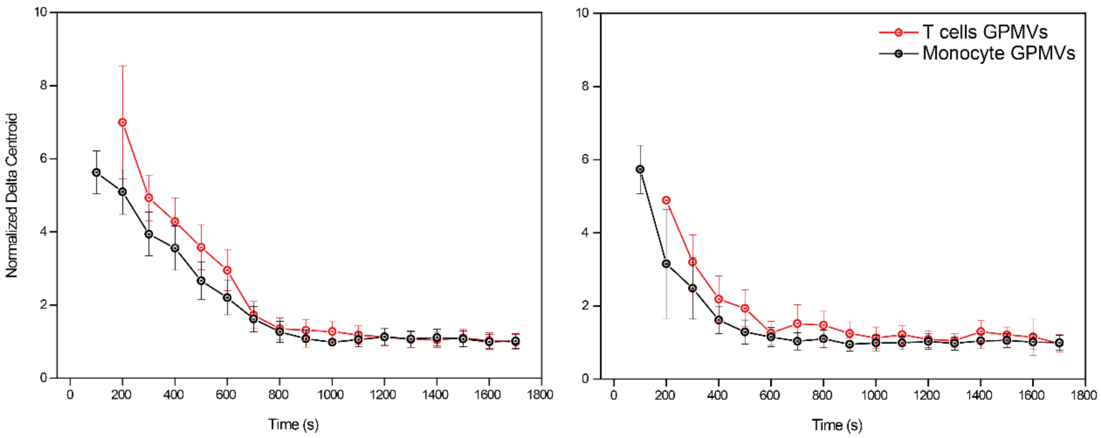

Figure S14. Data for technical repeats of R18 spatial distribution kinetic profiles in GPMVs. Kinetic profiles of R18 Δ^{norm}_{xy} after incubation of R18-labeled EVs with either T cell-derived (red) or monocyte-derived (black) GPMVs.

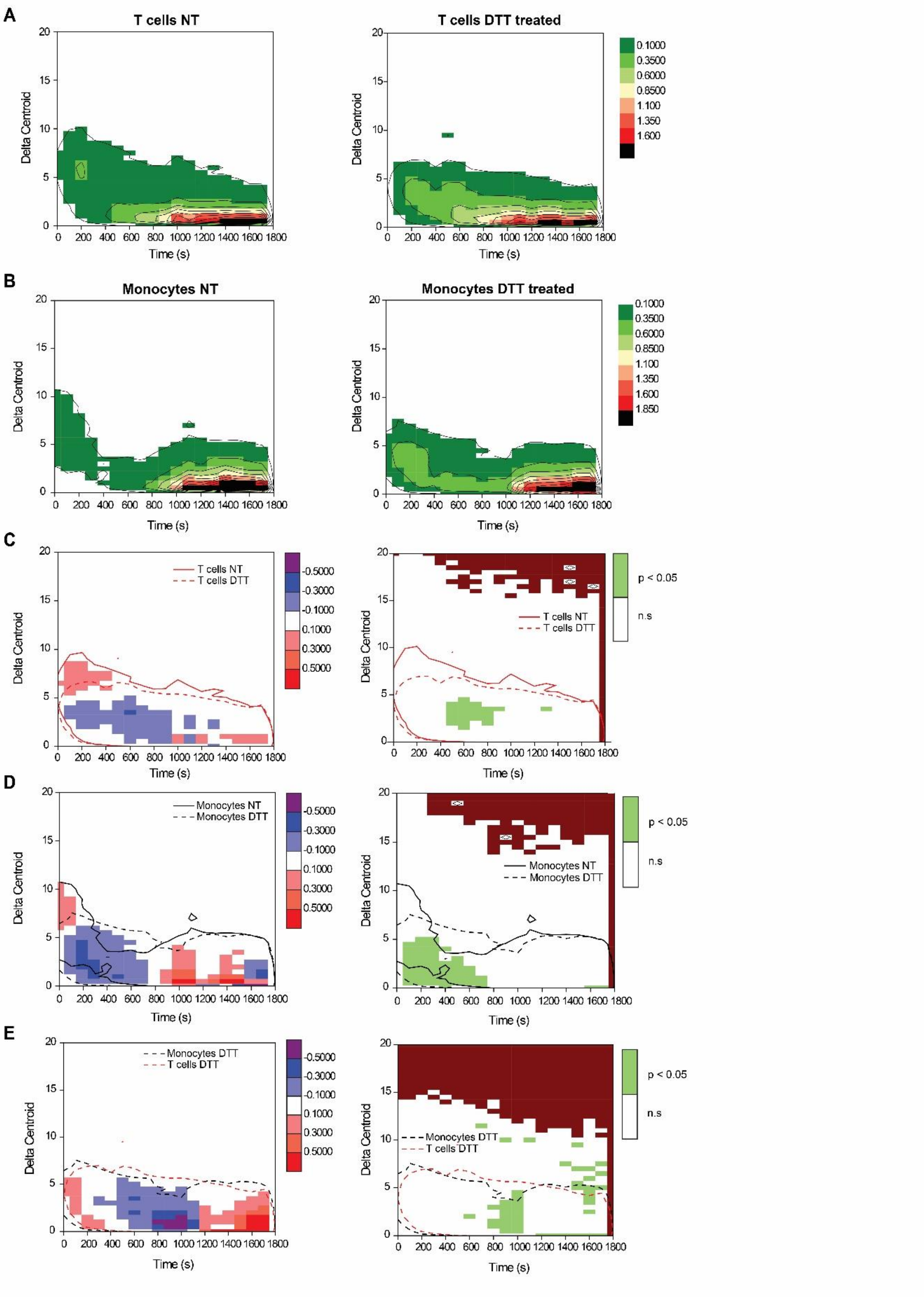

A
T cells NT
T cells DTT treated
0.1000
0.3500
0.6000
0.8500
1.100
1.350
1.600
B
Monocytes NT
Monocytes DTT treated
0.1000
0.3500
0.6000
0.8500
1.100
1.350
1.600
1.850
C
T cells NT
T cells DTT
-0.5000
-0.3000
-0.1000
0.1000
0.3000
0.5000
p < 0.05
n.s
D
Monocytes NT
Monocytes DTT
-0.5000
-0.3000
-0.1000
0.1000
0.3000
0.5000
p < 0.05
n.s
E
Monocytes DTT
T cells DTT
-0.5000
-0.3000
-0.1000
0.1000
0.3000
0.5000
p < 0.05
n.s
Delta Centroid
Time (s)

Figure S15. Uptake kinetics of R18-labeled *Pf*-derived EVs into monocytes and T cells following DTT treatment. (A) Heat map of average Δxy relative frequency after incubation of R18-labeled EVs with either untreated (left) or DTT-treated (right) T cells. **(B)** Heat map of average Δxy relative frequency after incubation of R18-labeled EVs with either untreated (left) or DTT-treated (right) monocytes. **(C)** Heat map of relative frequency difference (left) and the statistical significance (right) between untreated (red line) and DTT-treated (red dashed line) T cells. **(D)** Heat map of relative frequency difference (left) and the statistical significance (right) between untreated (black line) and DTT-treated (black dashed line) monocytes. **(E)** Heat map of relative frequency difference (left) and the statistical significance (right) between DTT-treated monocytes (black dashed line) and DTT-treated T cells (red dashed line). In all panels, outlines indicate the isofrequency region at a relative frequency of 0.1% for each condition. Statistical significance was calculated using one-way ANOVA; green indicates statistical significance ($p < 0.05$), white indicates no significance (n.s.), and brown indicates statistical test no*t* applicable. Data shown in all panels were obtained from 3 independent biological repeats.

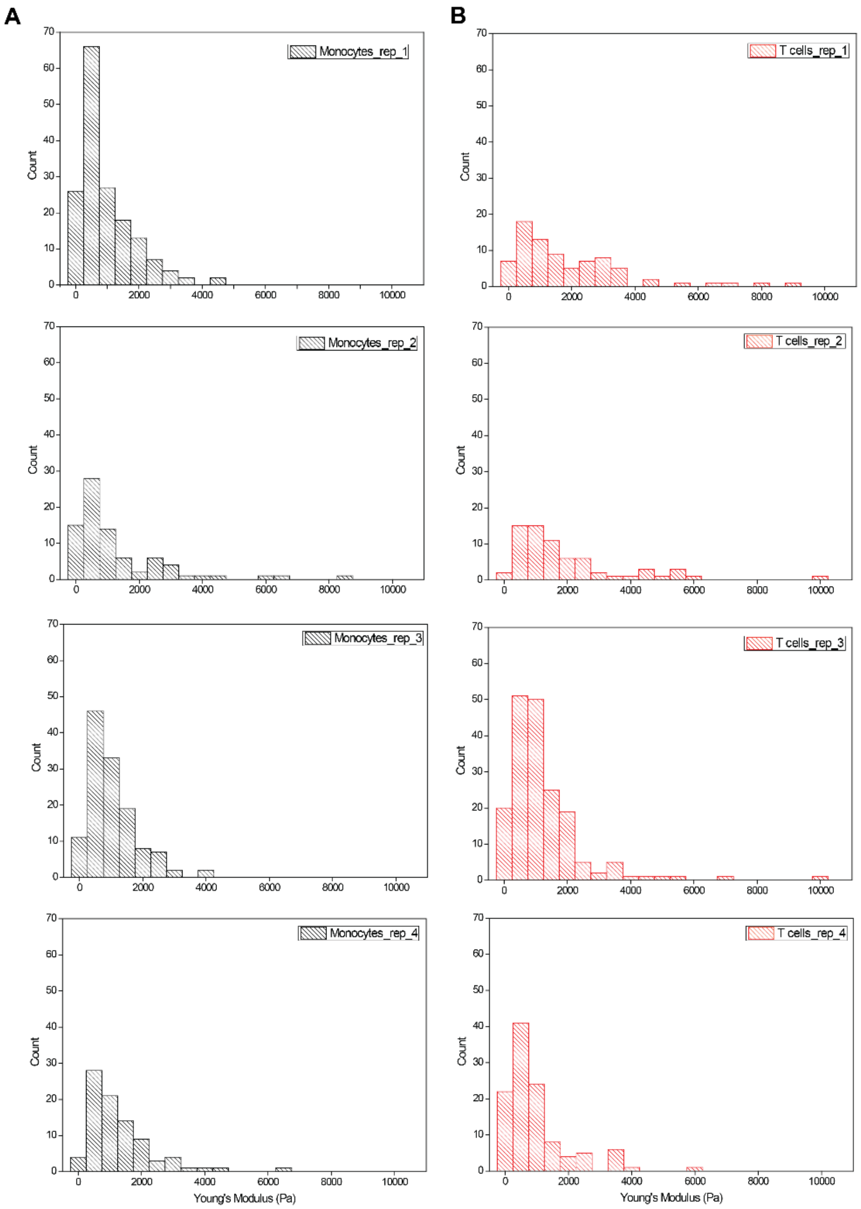

Figure S16. Data from technical repeats of AFM measurements of GPMV mechanical deformability. Young modulus distribution (bin width 500 Pa) of intact GPMVs obtained from **(A)** monocytes and **(B)** T cells.

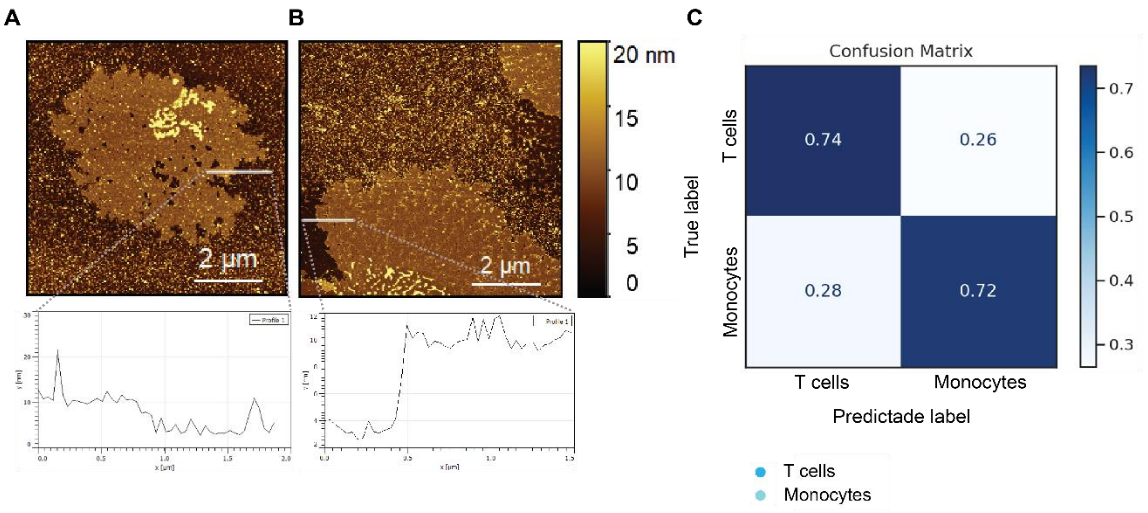

Figure S17. Machine-learning assisted analysis of AFM force curves reveals distinct behaviors between T cell- and monocyte-derived GPMVs. (A, B) Representative AFM images (as shown in Figure 6) and relative height profile measure via AFM (white line) for supported membranes obtained for **(A)** monocyte-detived and **(B)** T cell-derived GPMVs. **(C)** Confusion matrix of probability of true positives, true negatives, false positives, and false negatives in puncture data.

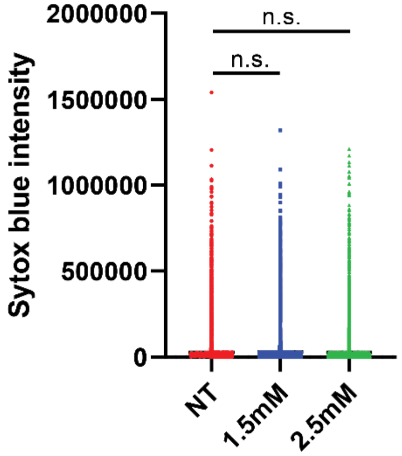

Figure S18. Viability of cholesterol-depleted T cells. Untreated T cells and T cells treated with1.5 mM and 2.5 mM mβCD were labeled with Sytox blue viability dye and signal was quantified using IFC. Statistical significance was calculated using one way ANOVA (n.s., not significant).

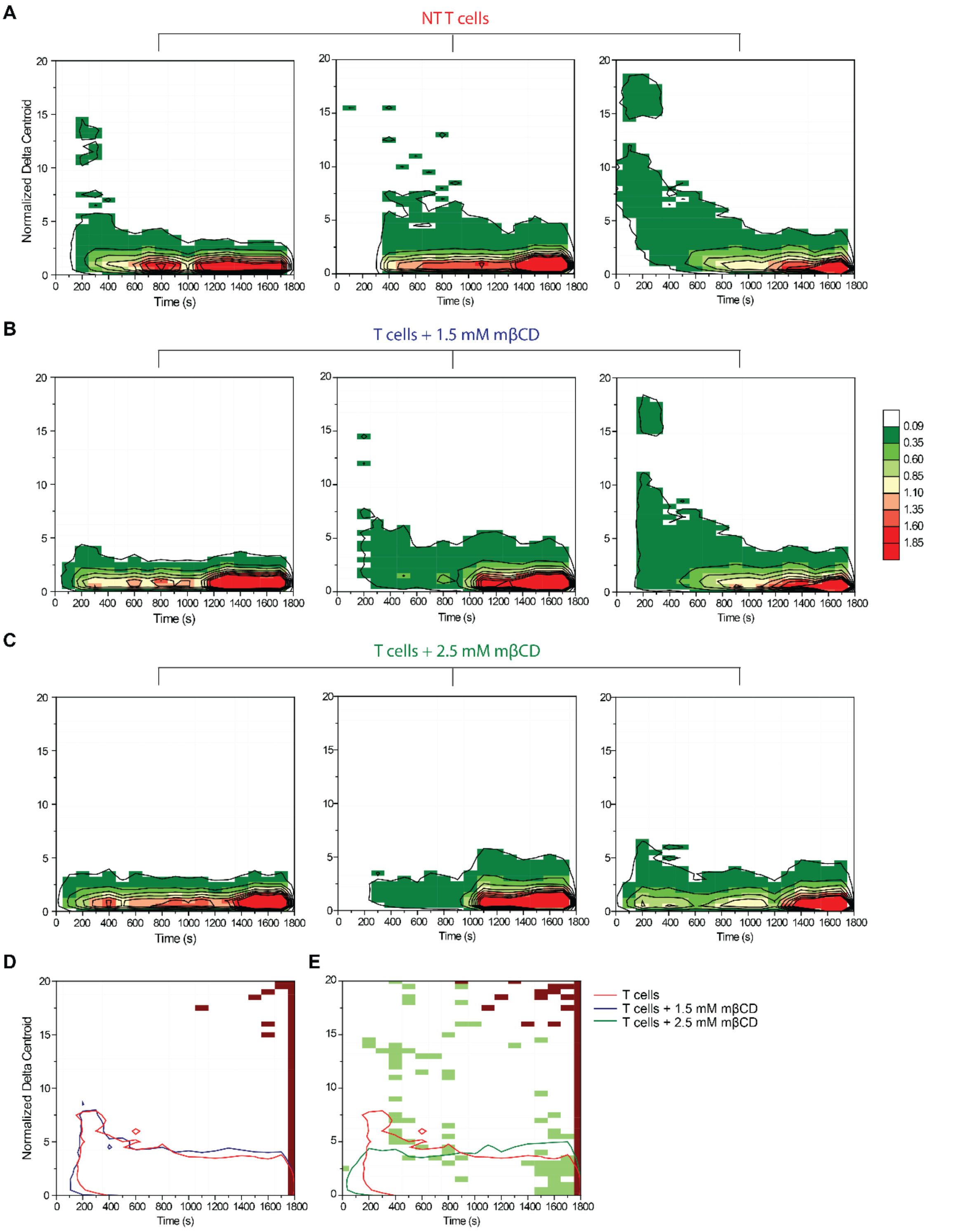

Figure S19. Data for technical repeats of R18 spatial distribution in cholesterol-depleted T cells. (A-C) Heat maps of Δ^{norm}_{xy} relative frequencies after incubation of R18-labeled EVs with **(A)** untreated T cells, **(B)** T cells treated with 1.5 mM mβCD, and **(C)** with T cells treated with 2.5 mM mβCD. **(D, E)** Heat maps displaying the statistical significance between untreated T cells and T cells treated with **(D)** 1.5 mM mβCD

and **(E)** 2.5 mM mβCD. In all panels, outlines indicate the isofrequency region at relative frequency 0.09% for either untreated T cells (red line), T cells treated with 1.5 mM mβCD (navy), or T cells treated with 2.5 mM mβCD (olive). Statistical significance was calculated using one way ANOVA; green indicates statistical significance ($p < 0.05$), white indicates no significance (n.s.), and brown indicates statistical test no*t* applicable. Data in all panels were obtained from 3 independent biological repeats.

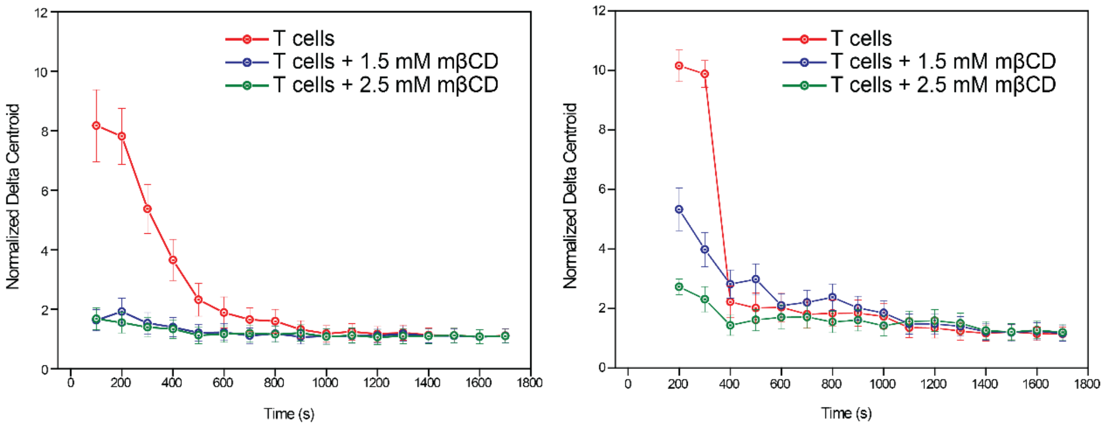

Figure S20. Data for technical repeats of R18 spatial distribution kinetic profiles of cholesterol depletion. Kinetic profiles of Δ^{norm}_{xy} after incubation of R18-labeled EVs with either untreated T cells (red), T cells treated with 1.5 mM mβCD (navy), or T cells treated with 2.5 mM mβCD (olive).
